# Possible Role of *MADS AFFECTING FLOWERING 3* and *B-BOX DOMAIN PROTEIN 19* in Flowering Time Regulation of Arabidopsis Mutants with Defects in Nonsense-Mediated mRNA Decay

**DOI:** 10.3389/fpls.2017.00191

**Published:** 2017-02-14

**Authors:** Zeeshan Nasim, Muhammad Fahim, Ji Hoon Ahn

**Affiliations:** ^1^Creative Research Initiatives, Department of Life Sciences, Korea UniversitySeoul, South Korea; ^2^Genetic Resources Conservation Lab, Institute of Biotechnology and Genetic Engineering, University of AgriculturePeshawar, Pakistan

**Keywords:** *UPF1*, *UPF3*, NMD, flowering, Arabidopsis

## Abstract

Eukaryotic cells use nonsense-mediated mRNA decay (NMD) to clear aberrant mRNAs from the cell, thus preventing the accumulation of truncated proteins. In Arabidopsis, two UP-Frameshift (UPF) proteins, UPF1 and UPF3, play a critical role in NMD. Although deficiency of *UPF1* and *UPF3* leads to various developmental defects, little is known about the mechanism underlying the regulation of flowering time by NMD. Here, we showed that the *upf1-5* and *upf3-1* mutants had a late-flowering phenotype under long-day conditions and the *upf1-5 upf3-1* double mutants had an additive effect in delaying flowering time. RNA sequencing of the *upf* mutants revealed that *UPF3* exerted a stronger effect than *UPF1* in the *UPF*-mediated regulation of flowering time. Among genes known to regulate flowering time, *FLOWERING LOCUS C* (*FLC*) mRNA levels increased (up to 8-fold) in *upf* mutants, as confirmed by qPCR. The *upf1-5, upf3-1*, and *upf1-5 upf3-1* mutants responded to vernalization, suggesting a role of *FLC* in delayed flowering of *upf* mutants. Consistent with the high *FLC* transcript levels and delayed flowering in *upf* mutants, levels of *FLOWERING LOCUS T* (*FT*) and *SUPPRESSOR OF OVEREXPRESSION OF CONSTANS 1* (*SOC1*) mRNAs were reduced in the *upf* mutants. However, RNA-seq did not identify an aberrant *FLC* transcript containing a premature termination codon (PTC), suggesting that *FLC* is not a direct target in the regulation of flowering time by NMD. Among flowering time regulators that act in an *FLC*-dependent manner, we found that *MAF3, NF-YA2, NF-YA5*, and *TAF14* showed increased transcript levels in *upf* mutants. We also found that *BBX19* and *ATC*, which act in an *FLC*-independent manner, showed increased transcript levels in *upf* mutants. An aberrant transcript containing a PTC was identified from *MAF3* and *BBX19* and the levels of the aberrant transcripts increased in *upf* mutants. Taking these results together, we propose that the late-flowering phenotype of *upf* mutants is mediated by at least two different pathways, namely, by *MAF3* in an *FLC*-dependent manner and by *BBX19* in an *FLC*-independent manner.

## Introduction

The survival of plant species largely depends on successful seed production via the formation of flowers; therefore, plants have evolved a complex mechanism to ensure successful reproduction (Huijser and Schmid, 2011), including a complicated genetic network that integrates endogenous and environmental cues to modulate the timing of the floral transition. At least 306 genes and eight genetic pathways affect flowering, including the photoperiod, autonomous, vernalization, ambient temperature, and gibberellic acid-dependent pathways (Bernier and Périlleux, 2005; Bouche et al., 2016).

*FLOWERING LOCUS C* (*FLC*) encodes a MADS-box transcription factor that binds to over 500 target sites in Arabidopsis and regulates genes involved in different developmental processes (Deng et al., 2011). FLC negatively regulates flowering (Michaels and Amasino, 1999) by repressing the expression of *FLOWERING LOCUS T* (*FT*) and *SUPPRESSOR OF OVEREXPRESSION OF CONSTANS 1* (*SOC1*; Searle et al., 2006), thereby preventing precocious flowering. *FLC* mRNA levels inversely correlate with the timing of flowering (Michaels and Amasino, 1999; Sheldon et al., 1999). The *flc* mutants show early flowering, whereas *FLC* overexpression causes very late flowering (Hepworth et al., 2002). Several pathways affect *FLC* expression, including the *FRIGIDA* (*FRI*) pathway, which activates *FLC* expression, the autonomous pathway, which negatively regulates *FLC*, and the vernalization pathway, which epigenetically silences *FLC* in response to prolonged cold. In vernalization, the expression of *FLC* is silenced through histone methylation and chromatin modification (Bastow et al., 2004; Michaels, 2009). *FLC* expression is regulated by various classes of proteins. For example, *MADS AFFECTING FLOWERING 3* (MAF3), a close homolog of FLC, directly interacts with FLC (Gu et al., 2013) and negatively regulates flowering at low temperatures (Suter et al., 2014). MAF3 also represses *FT* and *SOC1* expression through direct binding to their genomic loci (Gu et al., 2013). *FLC* is positively regulated by the members of the *NUCLEAR FACTOR Y* family, including *NF-YA2* and *NF-YA5* (Xu et al., 2013), which encode CCAAT-binding transcription factors.

In the regulation of flowering, important downstream targets of FLC include *FT and SOC1. FT* is considered the long-sought florigen (Zeevaart, 2008) and promotes the floral transition by directly interacting with FD to up-regulate the flower meristem identity gene *APETALA1 (AP1*) to specify the fate of floral cells (Abe et al., 2005). *SOC1* is a floral activator that encodes a conserved MADS box protein (Lee et al., 2000). *SOC1* mainly regulates *LEAFY* (*LFY*), another flower meristem identify gene, to induce floral initiation (Lee et al., 2000). The *ft* and *soc1* mutants exhibit a delayed flowering phenotype (Kobayashi et al., 1999; Borner et al., 2000), indicating their importance in the determination of the timing of flowering. Among several regulators that regulate *FT* expression, B-BOX DOMAIN PROTEIN 19 (BBX19) indirectly regulates *FT* expression by interacting with CONSTANS (CO) and preventing CO from inducing *FT* expression (Wang et al., 2014). Although *bbx19* mutation causes very weak early flowering, overexpression of *BBX19* causes a delayed flowering phenotype (Wang et al., 2014), suggesting that BBX19 acts as a floral repressor. *Arabidopsis thaliana CENTRORADIALIS* homolog (*ATC*) is a floral regulator that represses flowering upon overexpression (Mimida et al., 2001). ATC and FT can interact with FD and affect the expression of *AP1* (Huang et al., 2012). *ATC* overexpression causes late flowering in plants grown under both long day (LD) and short day (SD) conditions (Mimida et al., 2001). However, *atc* mutants exhibit an early flowering phenotype only under SD conditions (Mimida et al., 2001; Huang et al., 2012), suggesting the enhanced ability of ATC to compete with FT for FD interaction, when *FT* expression levels are low.

Alternative splicing is an important mechanism for controlling gene expression, since it adds proteomic complexity from the limited number of genes encoded in the genome. However, alternative splicing can generate a wide variety of unproductive isoforms carrying a premature termination codon (PTC; Filichkin and Mockler, 2012). To avoid accumulation of potentially harmful truncated proteins, RNA surveillance mechanisms clear these isoforms from the cell (Hori and Watanabe, 2005; Kertesz et al., 2006; Yoine et al., 2006; Riehs et al., 2008; Kurihara et al., 2009; Dai et al., 2016). One of these mechanisms, nonsense-mediated mRNA decay (NMD) clears PTC-containing aberrant transcripts from cells in eukaryotes (Maquat, 2005). About 1–10% of genes are regulated by NMD in different organisms such as yeast, flies, mammals, and plants (He et al., 2003; Mendell et al., 2004; Rehwinkel et al., 2005; Kurihara et al., 2009). In plants, the NMD is triggered by the presence of PTCs in a transcript, long 3′ untranslated regions, or intron-containing 3′ untranslated regions (Kertesz et al., 2006; Kerényi et al., 2008; Nyikó et al., 2013). A study of 270 *Arabidopsis* genes revealed that among these NMD-triggering features in plants, the presence of a PTC is the most frequent target of NMD (Kalyna et al., 2012).

In Arabidopsis, homologs of *UPF1* (Arciga-Reyes et al., 2006), *UPF2* (Kerényi et al., 2008), *UPF3* (Hori and Watanabe, 2005), and *SMG7* (Riehs et al., 2008) play important roles in NMD. Arabidopsis UPF1 shares a 50–75% amino acid sequence similarity with its homologs in yeasts, humans, *Caenorhabditis elegans*, and *Drosophila*, suggesting their functional similarities (Arciga-Reyes et al., 2006). UPF1 is required for the rapid degradation of mRNAs containing both spliced and unspliced PTCs. UPF3 is also required for the suppression of aberrant mRNAs originating from alternative splicing, emphasizing the important role of UPF3 in plant NMD (Hori and Watanabe, 2005). Collectively, these findings indicate that UPF1 and UPF3 are indispensable for the decay of abnormal transcripts in plants (Arciga-Reyes et al., 2006). Previous studies reported that the *upf1* and *upf3* mutations cause various developmental defects, including late flowering (Arciga-Reyes et al., 2006), but little is known about the mechanism of the regulation of flowering time in NMD-deficient mutants in Arabidopsis.

Here, we report that the *upf1* and *upf3* mutations led to delayed flowering and their double mutants showed an additive effect in delaying flowering time. RNA-seq analysis showed that *FLC* mRNA levels increased in *upf* mutants. Consistent with this, *FT* and *SOC1* mRNA levels decreased in *upf* mutants, suggesting that FLC functions as a main component of the late-flowering phenotype of *upf* mutants. However, in *upf* mutants, our RNA-seq analysis did not find a PTC-containing transcript from *FLC*. Among the genes that regulate *FLC*, the transcript levels of *MAF3, NF-YA2, NF-YA5*, and *TAF14* increased in *upf* mutants and among the genes that regulate flowering time in an *FLC*-independent manner, the transcript levels of *ATC* and *BBX19* increased in *upf* mutants. Also, we found aberrant transcripts that contain a PTC from *MAF3* and *BBX19*. Therefore, we propose that at least two pathways mediate the late-flowering phenotype of NMD-deficient mutants: a pathway in which *MAF3* acts in an *FLC*-dependent manner and one in which *BBX19* acts in an *FLC*-independent manner.

## Materials and methods

### Plant materials, growth conditions, and flowering time measurement

The *upf1-5* (SALK_112922) and *upf3-1* (SALK_025175) mutants used in this study were previously described (Hori and Watanabe, 2005; Arciga-Reyes et al., 2006). We generated *upf1-5 upf3-1* double mutants by crossing *upf1-5* and *upf3-1* mutants and confirmed their homozygosity by PCR-based genotyping using AtUPF1-1F and AtUPF1-2R primers for *upf1-5* and AtUPF3-4F and AtUPF3-6R primers for *upf3-1* (Hori and Watanabe, 2005; Riehs-Kearnan et al., 2012; Supplementary Table 4). For RNA-seq and qPCR analyses, 8-day-old wild-type (WT) Columbia-0 (Col-0), *upf1-5, upf3-1*, and *upf1-5 upf3-1* double mutants grown at 23°C under standard LD conditions (16:8 h light: dark) were used. Total leaf numbers and days to flowering were used for the measurement of flowering time. Total leaf numbers were counted when the primary inflorescences reached about 5 cm. Flowering time data are presented as a box plot (Spitzer et al., 2014). In our box plots, the center lines show the medians and plus signs (+) show the mean value; box limits indicate the 25th and 75th percentiles as determined by R software; whiskers extend 1.5 times the interquartile range (IQR) from the 25th and 75th percentiles, and outliers that exceeded the 1.5X IQR are represented by ovals. The number of plants measured is shown above each genotype in the box plot.

### Vernalization treatment

The *upf1-5* and *upf3-1* single mutants and *upf1-5 upf3-1* double mutants were vernalized by incubating their imbibed seeds at 4°C for 4 weeks, prior to sowing. For a non-vernalized control, the *upf* mutants and WT Col-0 seeds were imbibed at 4°C for 4 days. The vernalization response was recorded as differences in flowering time between vernalized and non-vernalized mutants. The flowering time difference in response to vernalization was statistically assessed using Student's *t*-test.

### RNA sequencing (RNA-seq)

Plants were grown for 8 days on MS plates at 23°C under standard LD conditions (16:8 h light: dark). About 50–100 seedlings were harvested at Zeitgeber Time 16 (ZT16) and pooled for RNA extraction, which was done using the Plant RNA Purification Reagent (Invitrogen). RNA sequencing was performed with a single biological replication for each sample. Because we did a single RNA-seq experiment, we verified the results via qPCR by using the same growth conditions. For RNA sequencing, library preparation was performed with an Illumina TruSeq Stranded Total RNA Sample Prep kit (Illumina), according to the manufacturer's protocols. Briefly, after removal of rRNA from the total RNA (~700 ng) using the rRNA removal kit, the RNA was cleaned using RNA purification beads. After the rRNA was removed, the remaining RNA was fragmented using fragment mix (EPH) at 94°C for 6 min. The fragmented RNA was primed with random hexamers and transcribed to first-strand cDNA using reverse transcriptase and random primers at 25°C for 10 min, 42°C for 15 min, and then 70°C for 15 min. Then a replacement strand was synthesized by incorporating dUTP in place of dTTP to generate double-stranded cDNA using polymerase at 16°C for 1 h. After cleanup of cDNA using sample purification beads, a single “A” nucleotide was added to the 3′ ends of the blunt fragments using A-tailing mix reagent by incubating at 37°C for 30 min and then at 70°C for 5 min. Indexing adapters were ligated to the ends of the DNA fragments using ligation mix two reagent at 30°C for 10 min. After washing with sample purification beads twice, PCR was performed to enrich DNA fragments having adapter molecules on both ends. Thermocycler conditions were as follows: 95°C for 3 min, 8 cycles of: 98°C for 20 s, 60°C for 15 s, and 72°C for 30 min, with a final extension at 72°C for 5 min. Finally, quality and band size of libraries were assessed using Agilent 2100 Bioanalyzer (Agilent). Libraries were quantified by qPCR using CFX96 Real Time System (Bio-Rad). After normalization, sequencing of the prepared library was conducted as paired-end reads on an Illumina HiSeq2000 sequencer. The resulting raw RNA-seq data are available at NCBI Gene Expression Omnibus (GEO) database under the accession number GSE87851.

### Data analysis

The raw sequence reads were processed for removal of adapter sequences, followed by qualitative analysis of raw reads using FastQC (http://www.bioinformatics.babraham.ac.uk/projects/fastqc). The high-quality reads were then mapped against the *Arabidopsis thaliana* reference genome TAIR10 (downloaded from http://arabidopsis.org) using TopHat 2.0.6 (https://ccb.jhu.edu/software/tophat/index.shtml) and Bowtie 2 (http://bowtie-bio.sourceforge.net/bowtie2/index.shtml) using default parameters.

### Identification of differentially expressed genes (DEGs)

The resulting mapped reads were then assembled and normalized transcript abundances were determined using Cufflinks version 2.2.1 (Trapnell et al., 2012; http://cole-trapnell-lab.github.io/cufflinks) with default settings. Cuffdiff version 2.2.1 was used for the identification of DEGs between WT plants and *upf* mutants with default parameters, including a false discovery rate of 5% (Trapnell et al., 2012). The output of Cuffdiff was further analyzed using R package “CummeRbund” (Goff et al., 2012). Genes with fold-change (FC) values of two or more and FPKM (fragments per kilobase of transcript per million mapped reads) values of one or more were considered as differentially expressed genes.

### Selection of flowering time regulators and *FLC* regulators

To get insight into the flowering time changes seen in *upf* mutants, we studied expression of 306 flowering time regulator genes acquired from the Flowering Interactive Database (FLOR-ID; Bouche et al., 2016). For the analysis of genes that regulate *FLC*, we collected a total of 58 genes that potentially regulate *FLC* expression (Xu et al., 2013; Bouche et al., 2016; Supplementary Table 2) and analyzed their expression patterns. For representation of differential gene expression in *upf* mutants, three heatmaps were generated: one for overall DEGs found in *upf* mutants, a second for expression of all 306 flowering time regulators, and a third map for 58 genes that were identified as *FLC* regulators.

### Quantitative real-time PCR (qPCR) analysis

Quantitative real-time PCR (qPCR) was employed to validate the transcriptome data of the floral integrators and their regulators. Total RNA was extracted from 8-day-old Arabidopsis seedlings sampled at ZT16, using Plant RNA purification reagent (Invitrogen). The extracted RNA (~2 μg) was reverse transcribed into cDNA using the Transcriptor First Strand cDNA Synthesis kit (Roche). Expression analysis was performed using *SYBR Green I Master* mix (Roche) in a LightCycler 480 (Roche). The data were normalized against two stable reference genes, *PP2AA3* (*AT1G13320*) and a *SAND* family gene (*AT2G28390*) (Hong et al., 2010). All the qPCR data are presented as the mean of two biological replicates with three technical replicates each and the error bars indicate the standard deviation. Statistical significance of differences of gene expression levels between the samples was assessed by using Student's *t*-test. *P* < 0.05 were considered as significant. Information on the primers that were used for qPCR in this study is shown in Supplementary Table 4.

### Identification of potential target genes of NMD

For detection of potential NMD targets, we used the CummeRbund package to individually analyze each flowering time gene to identify genes that show accumulation of aberrant transcripts in NMD-deficient mutants. The shortlisted potential target transcripts were then detected using RT-PCR using *PP2AA3* as an internal control (Hong et al., 2010). Sequences of normal and aberrant transcripts from RNA-seq data were extracted and analyzed for the presence of PTCs between the normal and aberrant transcripts. Putative cDNA sequences of both normal and aberrant transcripts were translated *in silico* to determine the effect of the PTCs on the translated protein.

## Results

### The *upf* single and double mutants showed late flowering

**Figure 1:**
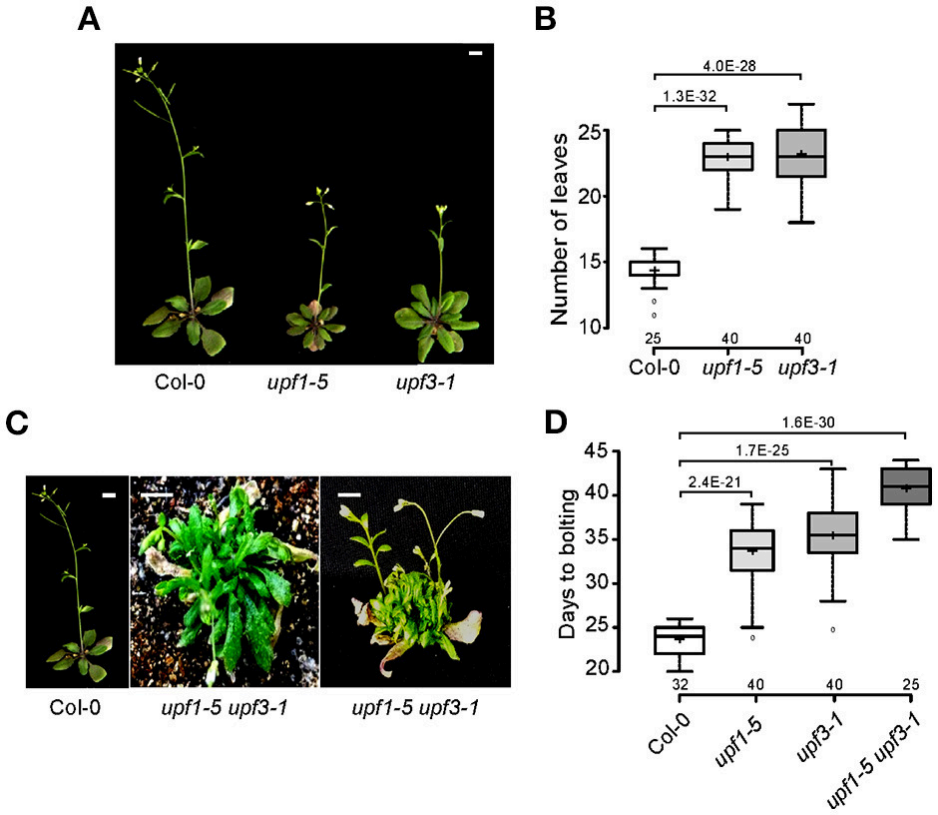
**Late flowering of *upf1-5, upf3-1*, and *upf1-5 upf3-1* mutants under LD conditions. (A)** Late-flowering phenotypes of *upf1-5* and *upf3-1* mutants grown at 23°C under LD conditions. Scale bar = 2 cm **(B)** Total leaf numbers of *upf1-5* and *upf3-1* mutants presented as a box plot (see Section Materials and Methods for further information on box plots). **(C)** The severely bushy phenotype of *upf1-5 upf3-1* double mutants with no well-defined leaves in the vegetative phase (middle) and multiple inflorescences generated from the axillary meristems in the reproductive phase (right). Scale bar = 2 cm (due to the late flowering of *upf1-5 upf3-1* mutants, pictures of wild-type Col-0 and double mutants were taken at different times. Col-0 plants were photographed at 5 weeks old and *upf1-5 upf3-1* double mutants were photographed at 8 weeks old). **(D)** Days to flowering of *upf1-5, upf3-1*, and *upf1-5 upf3-1* mutants presented as a box plot. *A t-test* was used to assess the statistical significance of flowering time between wild-type and *upf* mutants (*p*-values are mentioned for each group).

To investigate the effect of the loss of NMD function on flowering time, we measured flowering time of *upf1-5* and *upf3-1* single mutants and the *upf1-5 upf3-1* double mutants at 23°C under standard LD conditions. Both *upf1-5* and *upf3-1* single mutants flowered later than wild-type plants (Figures 1A,B), such that *upf1-5* and *upf3-1* mutants flowered with 22 ± 1.6 and 23 ± 2.3 leaves, respectively, consistent with a previous study that showed *upf1-5* and *upf3-1* mutants flowered late under both LD and SD conditions and had narrow, jagged rosette leaves (Arciga-Reyes et al., 2006). A previous study also reported that *upf3-1* mutants showed a more severe phenotype and grew more slowly than *upf1-5* mutants (Shi et al., 2012). We also generated *upf1-5 upf3-1* double mutants and measured their flowering time to investigate whether UPF1 and UPF3 act redundantly in flowering time. The *upf1-5 upf3-1* double mutants exhibited a severe bushy phenotype with no well-defined leaves in the vegetative phase (Figure 1C). Because multiple inflorescences emerged almost simultaneously from the axillary meristems of *upf1-5 upf3-1* double mutants after the transition to the reproductive phase (Figure 1C), we were not able to count the number of leaves of the primary inflorescence of *upf1-5 upf3-1* mutants; instead, we measured days to flowering. By this measure, the *upf1-5* and *upf3-1* mutants flowered later than wild-type plants under LD conditions. The *upf1-5* and *upf3-1* mutants flowered 33.5 ± 3.8 and 35.25 ± 3.6 days after germination (Figure 1D), respectively, whereas wild-type plants flowered 23.4 ± 1.7 days after germination. The *upf1-5 upf3-1* double mutants flowered 40.6 ± 2.4 days after germination, indicating that the double mutation had an additive effect in delaying flowering time. This flowering time analysis indicated that the *upf1-5* and *upf3-1* mutants are late flowering and their combined mutations have an additive effect on flowering.

### Differentially expressed genes (DEGs) in *upf* mutants

**Figure 2:**
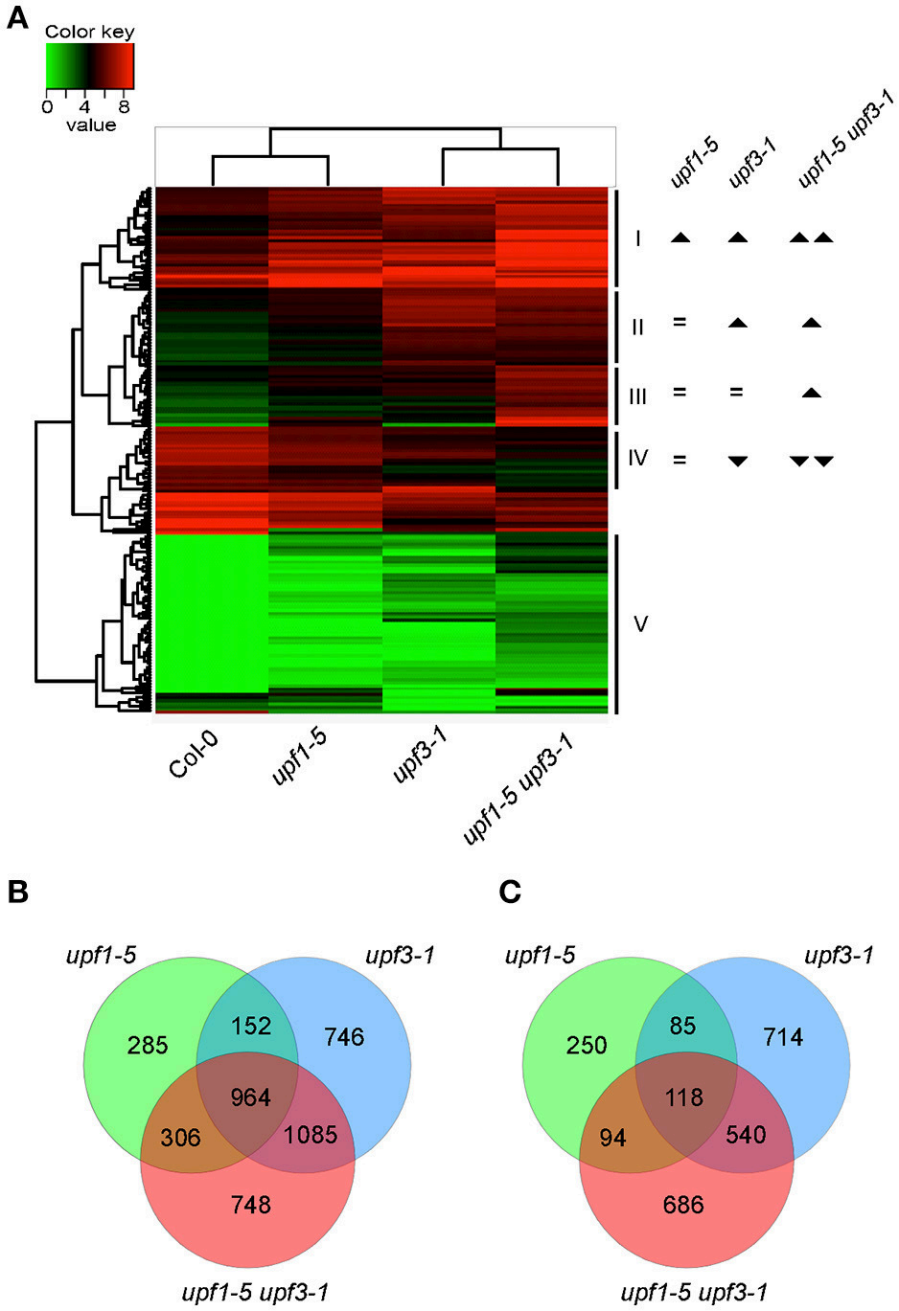
**Differentially expressed genes in *upf1-5, upf3-1*, and *upf1-5 upf3-1* mutants. (A)** A heatmap showing differentially expressed genes among *upf1-5, upf3-1, upf1-5 upf3-1*, and wild-type plants. Based on the differential gene expression, *upf1-5* mutants and wild-type plants grouped together, whereas *upf3-1* and *upf1-5 upf3-1* double mutants grouped together, indicating the high similarity between them. Upright triangle: up-regulation; inverted triangle: down-regulation. **(B)** Venn diagram of common and unique DEGs that were up-regulated in *upf1-5, upf3-1*, and *upf1-5 upf3-1* mutants as compared to wild-type plants. **(C)** Venn diagram of common and unique DEGs that were down-regulated in *upf1-5, upf3-1*, and *upf1-5 upf3-1* mutants as compared to wild-type plants. Note that the number of up-regulated genes was higher than down-regulated genes, suggesting the possible accumulation of NMD substrates in *upf* mutants.

To gain insights on the transcript levels of flowering time genes in *upf* mutants, we performed RNA sequencing (RNA-seq) using *upf1-5, upf3-1*, and *upf1-5 upf3-1* mutants with wild-type plants as a control (see Supplementary Table 1 for expression levels of all genes). Heat map analysis was performed to identify differentially expressed genes between *upf* mutants and wild-type plants (Figure 2A). Based on their expression patterns, we divided the DEGs into five major groups (Figure 2A). Group I included DEGs whose expression levels increased in both *upf1-5* and *upf3-1* mutants, but further increased in *upf1-5 upf3-1* mutants. Group II included DEGs whose expression levels increased in *upf3-1* mutants and *upf1-5 upf3-1* mutants. Group III included DEGs whose expression levels increased only in *upf1-5 upf3-1* mutants. Group IV included DEGs whose expression levels decreased in *upf3-1* mutants and further decreased in *upf1-5 upf3-1* mutants. The genes whose expression level changes were difficult to classify were placed in group V (Figure 2A). This heatmap analysis suggested that the expression patterns of DEGs in *upf3-1* mutants were more similar to *upf1-5 upf3-1* mutants than *upf1-5* mutants. Furthermore, scatter plot distribution of gene expression also revealed the similarities in DEGs between *upf3-1* single and *upf1-5 upf3-1* double mutants (Supplementary Figure 1).

We then selected individual DEGs after applying specific filters (genes with at least 2-fold difference in expression levels and FPKM value of 1 or more) from our RNA-seq data. The results showed that 2,254 genes were differentially expressed in *upf1-5* mutants (1,707 were up-regulated and 547 were down-regulated), 4,404 genes were differentially expressed in *upf3-1* mutants (2,947 were up-regulated and 1,457 were down-regulated; Figures 2B,C). In *upf1-5 upf3-1* mutants, 4,541 genes were differentially expressed (3,103 up-regulated and 1,438 down-regulated). A total of 964 genes were commonly up-regulated among *upf* single mutants and *upf1-5 upf3-1* double mutants. *upf1-5* and *upf1-5 upf3-1* double mutants shared 1,270 up-regulated genes, whereas *upf3-1* and *upf1-5 upf3-1* mutants shared 2,049 up-regulated genes (Figure 2B). In contrast, the *upf1-5* and *upf1-5 upf3-1* double mutants shared 212 down-regulated genes, whereas *upf3-1* and *upf1-5 upf3-1* mutants shared 658 down-regulated genes (Figure 2C). These indicated that in terms of number of DEGs, the *upf3-1* mutants were closer to the *upf1-5 upf3-1* mutants, compared with the *upf1-5* mutants. Considering that the *upf1-5 upf3-1* mutants showed an additive delayed flowering phenotype, these overall similarities in the number of DEGs between *upf3-1* mutants and *upf1-5 upf3-1* double mutants suggested that *UPF3* exerted a stronger effect than *UPF1* in the regulation of *UPF*-mediated flowering time.

To examine the biological functions of the DEGs, we performed Gene Ontology (GO) analysis using the ClueGO Cytoscape plug-in (Bindea et al., 2009). The “GO term fusion” option of ClueGO was used to reduce redundancy in GO terms. As expected, a variety of biological functions were affected by the NMD deficiency in *upf* mutants (Supplementary Figure 2). Most of the DEGs showed high enrichment for cellular metabolism of different biomolecules, metabolic process, gene expression regulation, transportation, protein localization, and response to different stimuli (Supplementary Figure 2), consistent with a previous finding (Rayson et al., 2012).

### Up-regulation of *FLC* in *upf* mutants

**Figure 3:**
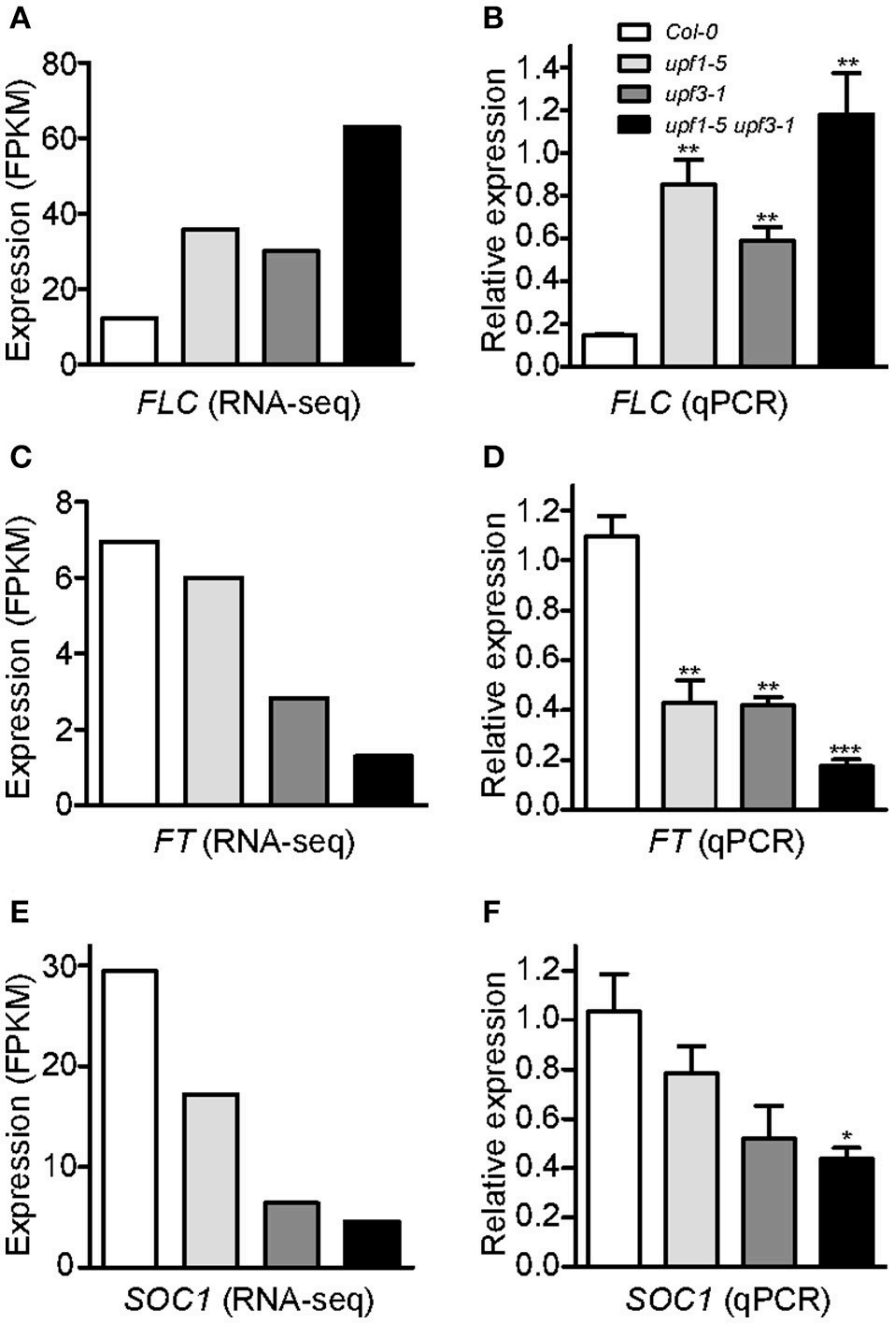
**An increase in *FLC* mRNA levels and a decrease of *FT* and *SOC1* mRNA levels in *upf* mutants. (A,B)** FPKM levels determined by RNA-seq **(A)** and relative expression levels determined by qPCR **(B)** of *FLC* in *upf1-5, upf3-1, upf1-5 upf3-1*, and wild-type plants. **(C,D)** FPKM levels determined by RNA-seq **(C)** and relative expression levels determined by qPCR **(D)** of *FT* in *upf1-5, upf3-1, upf1-5 upf3-1*, and wild-type plants. **(E,F)** FPKM levels determined by RNA-seq **(E)** and relative expression levels determined by qPCR **(F)** of *SOC1* in *upf1-5, upf3-1, upf1-5 upf3-1*, and wild-type plants.

To identify potential candidate genes responsible for the delayed flowering of *upf* mutants, we looked for DEGs related to the regulation of flowering time. Among such genes (Supplementary Figure 3), we found that the mRNA levels of *FLOWERING LOCUS C* (*FLC*), a floral repressor that is important for the vernalization response (Michaels and Amasino, 1999), increased in *upf1-5, upf3-1*, and *upf1-5 upf3-1* mutants (Figure 3A, Supplementary Table 2). Our RNA-seq data showed that *FLC* mRNA levels increased by 2.92-fold in *upf1-5* mutants and by 2.45-fold in *upf3-1* mutants, whereas *FLC* mRNA levels increased by more than 5-fold in *upf1-5 upf3-1* double mutants, compared to wild-type plants, indicating that *UPF1* and *UPF3* have an additive effect in regulating *FLC* expression (Figure 3A, Supplementary Table 2).

As our global scale RNA-seq analysis revealed the elevated levels of *FLC* in the upf mutants that we tested (Figure 3A, Supplementary Table 2), we performed qPCR analyses to confirm the finding. *FLC* mRNA expression levels increased in *upf1-5* and *upf3-1* mutants (5.8- and 4-fold, respectively; Figure 3B). This suggested a role of *FLC* in delayed flowering of NMD-deficient mutants. Consistent with the further delayed flowering seen in *upf1-5 upf3-1* mutants (Figure 1), *FLC* mRNA levels further increased in *upf1-5 upf3-1* mutants (8.1-fold; Figure 3B).

Consistent with the increased levels of *FLC*, we also found that the mRNA levels of *FLOWERING LOCUS T* (*FT*; Kardailsky et al., 1999) and *SOC1* (Lee et al., 2000), which were reported as major targets of *FLC* (Helliwell et al., 2006), decreased (Supplementary Table 2). Our RNA-seq analysis showed down-regulation of *FT* in *upf1-5* and *upf3-1* mutants (1.1 and 2.63-fold, respectively). In the *upf1-5 upf3-1* double mutants, the mRNA levels of *FT* decreased by 5.1-fold, suggesting that the *upf1* and *upf3* mutations showed an additive effect (Figure 3C, Supplementary Table 2). Similar to *FT*, the mRNA levels of *SOC1*, another important floral integrator that is repressed directly by FLC (Hepworth et al., 2002), were reduced in *upf1-5* and *upf3-1* single mutants (1.7- and 4.5-fold, respectively), whereas the *upf1-5 upf3-1* double mutants showed a 6.3-fold decrease (Figure 3E, Supplementary Table 2). Our qPCR results were consistent with our RNA-seq data that showed decreased levels of *FT* (Figure 3D) and *SOC1* mRNAs (Figure 3F) in *upf* mutants. We also analyzed *SHORT VEGETATIVE PHASE* (*SVP*) expression levels, a*s* FLC interacts with SVP to synergistically repress flowering (Li et al., 2008). However, we did not find apparent changes in *SVP* mRNA levels in *upf* mutants (Supplementary Figure 4). These results showed that *FLC* mRNA levels increased, whereas *FT* and *SOC1* mRNA levels decreased in *upf* mutants, raising the possibility that *FLC* up-regulation represses *FT* and *SOC1* expression in *upf* mutants, which eventually causes late flowering in *upf* mutants.

### *upf* mutants were vernalization-responsive

**Figure 4:**
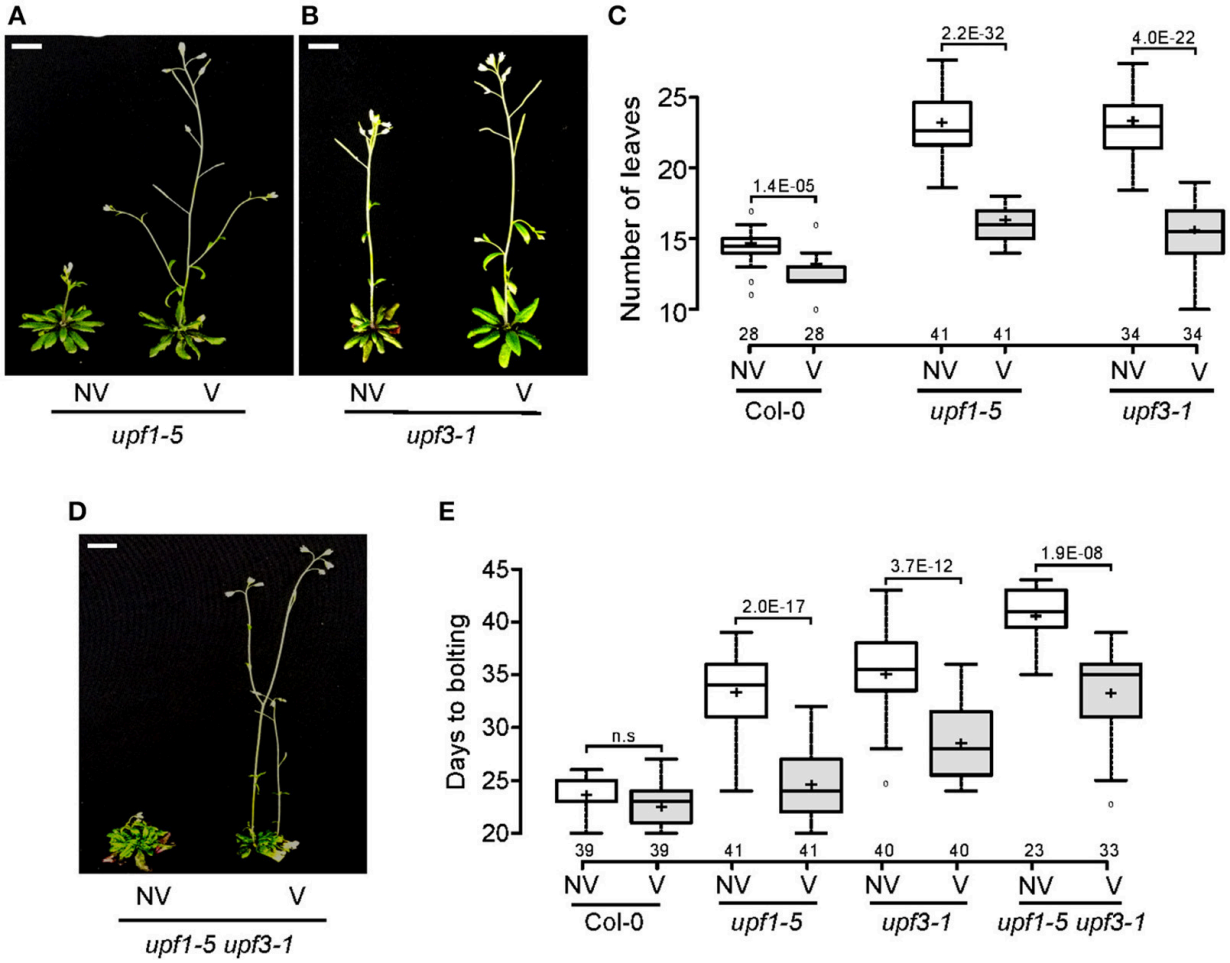
**Vernalization response of *upf1-5*, *upf3-1*, and *upf1-5 upf3-1* mutants. (A,B)** Acceleration of flowering by vernalization in *upf1-5* **(A)** and *upf3-1* mutants **(B)**. NV: non-vernalized, V: vernalized. Scale bar = 2 cm **(C)** Box plot showing total leaf numbers of vernalized/non-vernalized *upf1-5* and *upf3-1* mutants. Both *upf1-5* and *upf3-1* mutants flowered earlier in response to vernalization treatment. **(D)** Vernalization response of *upf1-5 upf3-1* double mutants. NV: non-vernalized, V: vernalized. **(E)** Box plot showing days to flowering of vernalized/non-vernalized *upf1-5, upf3-1*, and *upf1-5 upf3-1* mutants. A *t-test* was used to assess the statistical significance of flowering time difference in response to vernalization (*p-value* s mentioned for each group).

After confirming the elevated levels of *FLC* in *upf* mutants, we next investigated whether *FLC* mediates the late-flowering phenotype of *upf* mutants. For this purpose, we analyzed whether vernalization treatment accelerated flowering of *upf* mutants, as *FLC*-mediated repression of flowering can be recovered by prolonged cold treatment (Michaels and Amasino, 1999; Sheldon et al., 1999). We transferred *upf* mutant seeds to 4°C for 4 weeks for vernalization treatment. Flowering time measurement indicated that vernalization accelerated the flowering of *upf1-5* and *upf3-1* mutants. Vernalized *upf1-5* mutants flowered with 15.0 ± 1.5 leaves, whereas non-vernalized *upf1-5* mutants flowered with 22.7 ± 2.2 leaves (Figures 4A,C). Similarly, vernalized *upf3-1* mutants flowered with 16.3 ± 1.0 leaves, whereas non-vernalized *upf3-1* mutants flowered with 23.3 ± 2.1 leaves (Figures 4B,C). The non-vernalized Col-0 plants flowered with 14.3 ± 1.3 leaves and the vernalized Col-0 plants flowered with 12.4 ± 1.2 leaves (Figure 4C). These observations confirmed that wild-type Col-0 plants, which contain a non-functional *FRI* allele (Lee and Amasino, 1995), showed weak responsiveness to vernalization treatment. As leaf numbers could not be counted for *upf1-5 upf3-1* mutants, we also measured days to flowering of vernalized *upf1-5 upf3-1* mutants under LD conditions to determine the vernalized response of *upf1-5 upf3-1* mutants. The vernalized wild-type plants flowered 22.4 ± 2.3 days after germination, compared to the non-vernalized wild-type plants, which flowered 23.7 ± 1.6 days after germination. Vernalized *upf1-5* mutants flowered 24.7 ± 3.5 days after germination, whereas non-vernalized *upf1-5* mutants flowered 33.4 ± 3.8 days after germination (Figure 4E). Similarly, vernalized *upf3-1* mutants flowered 28.1 ± 3.4 days after germination, whereas non-vernalized *upf3-1* mutants flowered 35.6 ± 3.6 days after germination. In addition, vernalized *upf1-5 upf3-1* mutants flowered 33 ± 4.3 days after germination, whereas non-vernalized *upf1-5 upf3-1* mutants flowered 40.7 ± 2.4 days after germination (Figures 4D,E). These analyses indicated that vernalized *upf1-5, upf3-1*, and *upf1-5 upf3-1* mutants flowered ~9, 7.5, and 7.7 days earlier than non-vernalized mutants, respectively. These observations suggested that *FLC* repression by vernalization likely accelerated flowering in *upf* mutants, suggesting a key role of *FLC* in the late flowering of *upf* mutants.

### *UPF*-mediated regulation of flowering time may target *FLC* indirectly

Although it seemed that the late-flowering phenotype of *upf* mutants was largely mediated by *FLC*, we next looked for an aberrant *FLC* transcript that contains a PTC. In addition to AT5G10140.1, the predominant transcript that produces functional FLC protein, we found four alternatively spliced isoforms of *FLC* in our RNA-seq (Supplementary Figure 5A). Among them, AT5G10140.2, AT5G10140.3, and AT5G10140.4 were already reported. We found an unreported alternatively spliced isoform and named it AT5G10140.5. AT5G10140.5 had a similar structure to the fourth splice variant of *FLC* (i.e., AT5G10140.4), except for its untranslated regions (UTRs), as the novel AT5G10140.5 transcript had longer 3′- and 5′-UTRs. We then measured the transcript levels of each alternatively spliced isoform. The abundance of these four isoforms of *FLC* was not apparently altered in *upf* mutants (Supplementary Figure 5B). Moreover, we could not find a PTC from any of the alternative spliced isoforms. These results suggested that *FLC* is not a direct target of NMD in the regulation of flowering time in *upf* mutants.

### Expression of *MAF3, TAF14*, and *NF-YA2/5*, positive regulators of *FLC*, was up-regulated in *upf* mutants

**Figure 5:**
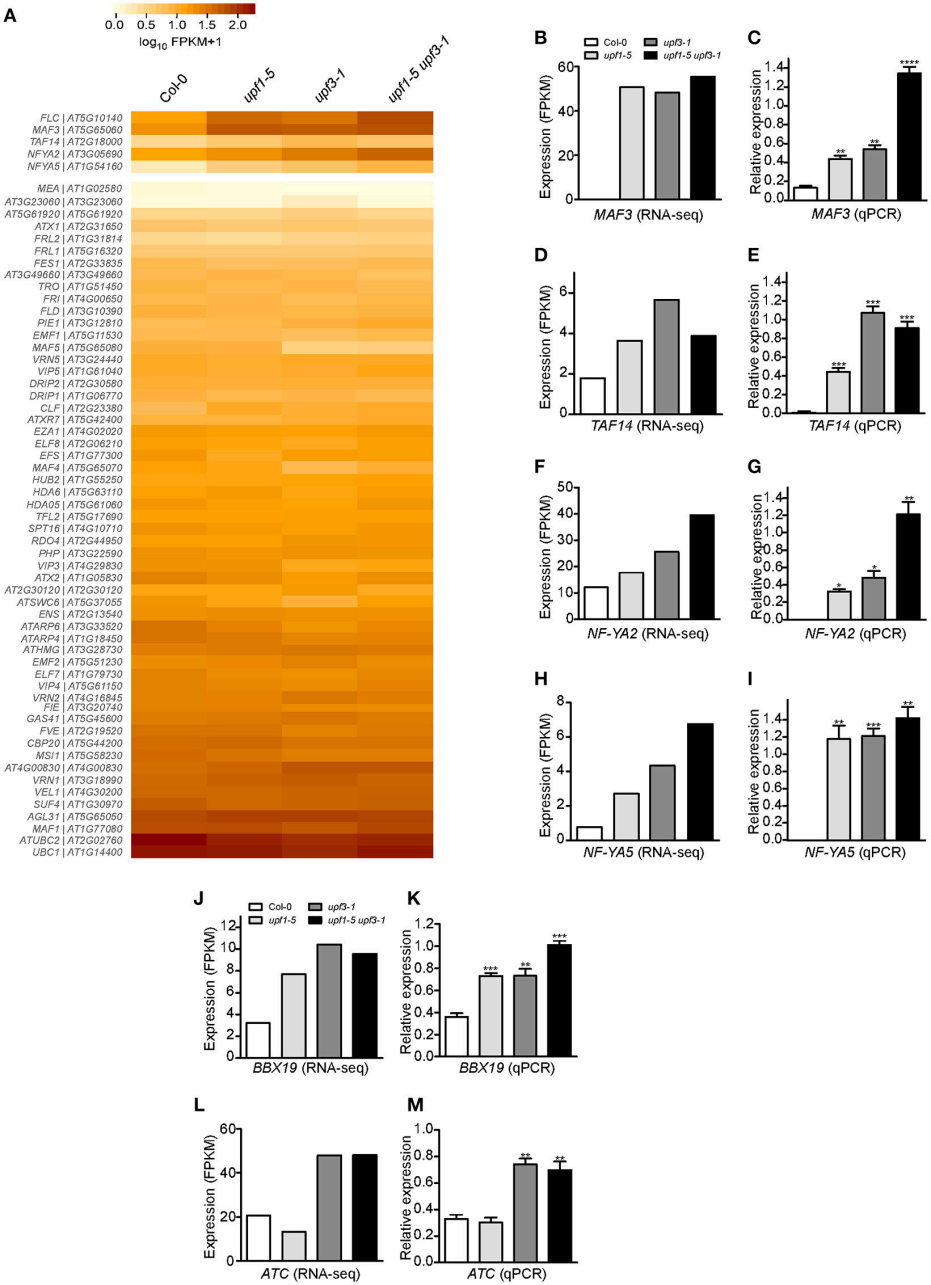
**Expression of *FLC* and its regulators in *upf* mutants. (A)** Heatmap shows the expression of 58 genes that possibly regulate *FLC* expression. *MAF3, TAF14, NF-YA2*, and *NF*-*YA5*, which showed expression patterns similar to that of *FLC*, are shown separately. **(B,C)** FPKM levels determined by RNA-seq **(B)** and relative expression levels determined by qPCR **(C)** of *MAF3* in *upf1-5, upf3-1, upf1-5 upf3-1*, and wild-type plants. **(D,E)** FPKM levels determined by RNA-seq **(D)** and relative expression levels determined by qPCR **(E)** of *TAF14* in *upf* mutants. *TAF14* mRNA levels increased in *upf* single and double mutants, as detected by RNA-seq **(D)** and confirmed by qPCR **(E)**. **(F,G)** FPKM levels determined by RNA-seq **(F)** and relative expression levels determined by qPCR **(G)** of *NF-YA2* in *upf* mutants. **(H,I)** FPKM levels determined by RNA-seq **(H)** and relative expression levels determined by qPCR **(I)** of *NF-YA5* in *upf* mutants. **(J,K)** FPKM levels determined by RNA-seq **(J)** and relative expression levels determined by qPCR **(K)** of *BBX19* in *upf* mutants. **(L,M)** FPKM levels determined by RNA-seq **(L)** and relative expression levels determined by qPCR **(M)** of *ATC* in *upf* mutants.

As *FLC* may not be the direct target of *UPF*-mediated regulation of flowering time, we analyzed expression of genes that act upstream of *FLC*. For this purpose, we measured the transcript levels of 58 genes that regulate flowering time (Bouche et al., 2016) from our RNA-seq data (Figure 5A), to understand the positive regulation of *FLC* in the *upf* mutants. Detailed expression data for of all *FLC* regulators can be found in Supplementary Table 3. Among 58 genes that potentially regulate *FLC* expression, we found a few genes whose expression patterns in the mutants were similar to that of *FLC* (Figures 5B–I). They were: (1) *MADS AFFECTING FLOWERING 3* (*MAF3*), a homolog of *FLC* that inhibits flowering by up-regulating *FLC* expression and repressing *FT* and *SOC1* expression (Gu et al., 2013; Suter et al., 2014); (2) *TBP-ASSOCIATED FACTOR 14* (*TAF14*), which is also known as *HOMOLOG OF YEAST YAF9 B* (*YAF9B*) and promotes *FLC* expression through chromatin modification together with *AtYAF9A* (Zacharaki et al., 2012; Bieluszewski et al., 2015); and (3) *NF-YA2* and *NF-YA5*, which encode factors that may have dual functions (as a floral promoter and a floral repressor) in the regulation of flowering time (Xu et al., 2013; Hou et al., 2014).

RNA-seq analysis showed that mRNA levels of *MAF3* were high in *upf1-5* (2.9-fold that of wild type) and *upf3-1* (2.8-fold) mutants. The mRNA level of *MAF3* increased by 3.3-fold in *upf1-5 upf3-1* double mutants (Figure 5B, Supplementary Table 3). Similarly, qPCR analysis showed increased transcript levels of *MAF3* in *upf1-5* (3.3-fold) and *upf3-1* mutants (~4-fold; Figure 5C). The *upf1-5 upf3-1* double mutants showed a 10.2-fold increase, suggesting that both *upf* mutations had an additive effect on *MAF3* expression (Figure 5C). The mRNA levels of *TAF14* also increased in *upf1-5* (3.2-fold) and *upf3-1* (2.0-fold) mutants, as well as in *upf1-5 upf3-1* (2.1-fold) double mutants, (Figure 5D, Supplementary Table 3). The increased mRNA levels of *TAF14* revealed by qPCR (Figure 5E) were consistent with RNA-seq data. The RNA-seq data showed that the mRNA level of *NF-YA2* increased by 1.5- and 2.1-fold in *upf1-5* and *upf3-1* mutants, respectively, and was 3.3-fold higher in *upf1-5 upf3-1* double mutants (Figure 5F, Supplementary Table 3). The qPCR results agreed with the RNA-seq data of elevated *NF-YA2* mRNA levels (Figure 5G). The *upf1-5* and *upf3-1* mutants showed high levels of *NF-YA5* mRNA (3.5- and 5.6-fold increase, respectively), and *upf1-5 upf3-1* mutants showed an 8.7-fold increase in *NF-YA5* mRNA (Figure 5H, Supplementary Table 3), suggesting that *upf1* and *upf3* mutations contribute additively to *NF-YA5* mRNA levels in *upf1-5 upf3-1* double mutants. The qPCR analysis of *NF-YA2* also showed a similar expression pattern (Figure 5I).

### Expression of *BBX19* and *ATC*, genes that regulate flowering time in an *FLC*-independent manner, was up-regulated in *upf* mutants

We also analyzed the expression of genes that regulate flowering time in an *FLC-*independent manner to identify possible regulators of flowering time in NMD-deficient mutants. From our RNA-seq data, we found two floral repressors that showed consistent expression changes in *upf* mutants: *B-BOX DOMAIN PROTEIN 19* (*BBX19*), which encodes a floral repressor that interacts with CO, thereby preventing CO from inducing *FT* expression (Wang et al., 2014), and *ATC*, which encodes a floral repressor that interacts with FD to affect the expression levels of the floral meristem identity gene *AP1* (Mimida et al., 2001; Huang et al., 2012; Figures 5J,L). qPCR analysis revealed that *BBX19* expression was significantly up-regulated with a fold change of ~2 in *upf1-5* and *upf3-1* mutants and ~3-fold in *upf1-5 upf3-1* double mutants (Figure 5K). The transcript levels of *ATC* were similar between wild-type plants and *upf1-5* mutants (Figure 5M); however, the *ATC* expression levels were elevated over 2-fold in *upf3-1* and *upf1-5 upf3-*1 double mutants (Figure 5M). Thus, the elevated levels of *BBX19* and *ATC* in *upf* mutants suggested an *FLC*-independent contribution to the delayed flowering of *upf* mutants.

### *MAF3* and *BBX19* are potential targets of NMD in the determination of flowering time

**Figure 6:**
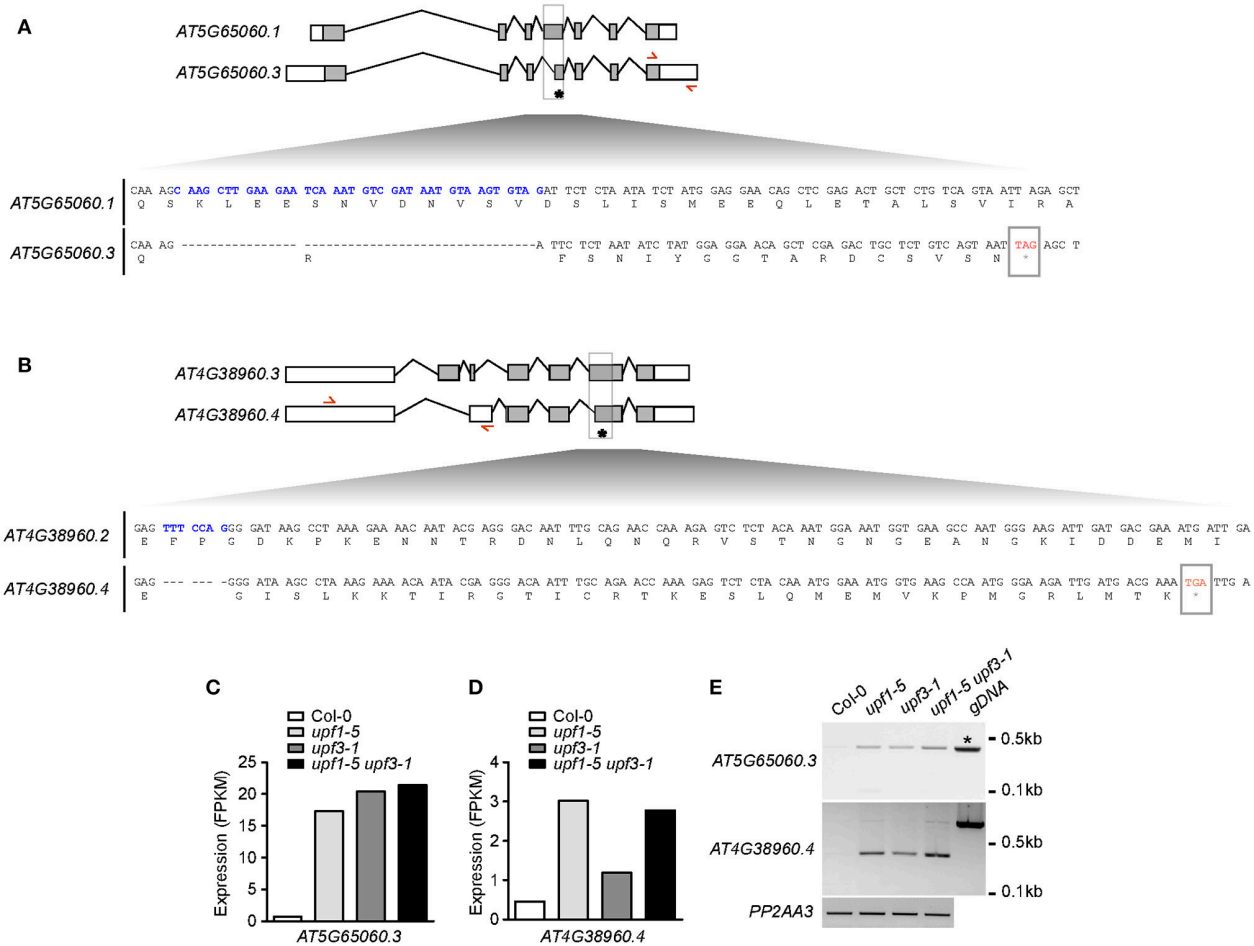
**Production of aberrant *MAF3* and *BBX19* transcripts containing PTCs in *upf* mutants. (A,B)** Comparison of the structures of a normal transcript (AT5G65060.1) and an aberrant transcript (AT5G65060.3) from *MAF3* **(A)** and comparison of the structures of a normal transcript (AT4G38950.3) and an aberrant transcript (AT4G38950.4) that we identified from *BBX19* **(B)** from our RNA-seq analysis. Each bottom panel shows deduced amino acid sequences from the normal transcript and the aberrant transcript containing a PTC, which is indicated by an asterisk. Sequences in blue indicate the cDNA fragment that is absent in the aberrant transcript. Red arrows indicate the binding sites of primers used to detect aberrant transcripts from *MAF3* and *BBX19*. Gray boxes indicate coding regions. **(C,D)** FPKM levels of the aberrant transcript of *MAF3* (AT5G65060.3) **(C)** and *BBX19* (AT4G38960.4) **(D)** in *upf* mutants. **(E)** Detection of AT5G65060.3 and AT4G38960.4 transcripts via RT-PCR. Note that an amplicon with the same size was amplified from genomic DNA (gDNA) from *MAF3* (asterisk). *PP2AA3* was used as an internal control.

To identify the direct targets of NMD in the regulation of flowering time, we analyzed the isoforms of all flowering time regulators. Among these genes, we found that *MAF3* and *BBX19* produced aberrantly spliced transcripts (Figures 6A,B). As these transcripts were not listed in TAIR, we then named them AT5G65060.3 (from *MAF3*) and AT4G38960.4 (from *BBX19*). The AT5G65060.3 transcript differed from AT5G65060.1, the predominant *MAF3* transcript, by the presence of longer 3′- and 5′-UTRs and the absence of 38 nucleotides within the 4th exon. The 38-nucleotide deletion caused a predicted out-of-frame splicing event, which created a PTC in the same exon, leading to formation of a truncated MAF3 protein (Figure 6A). The full-length alignment of the transcripts and the proteins can be found in Supplementary Figures 6, 7. The AT4G38960.4 transcript generated from *BBX19* had a long 5′-UTR, skipped the first two *BBX19* exons, and retained part of the third intron, like the AT4G38960.2 transcript, a splice variant of *BBX19*. Importantly, seven nucleotides were absent in the 6th exon of the AT4G38960.4 transcript, compared to AT4G38960.3, the normal functional transcript of *BBX19*. The 7-nucleotide deletion likely created a PTC in the 5th exon to generate truncated BBX19 proteins (Figure 6B, Supplementary Figure 9). Considering that the AT5G65060.3 and AT4G38960.4 transcripts had a PTC and long UTRs (Figures 6A,B, Supplementary Figures 6, 8), a characteristic of aberrant transcripts that are destined for NMD under normal circumstances, these AT5G65060.3 and AT4G38960.4 transcripts could be potential targets of *UPF*-mediated regulation of flowering time.

RNA-seq analysis showed that FPKM levels of AT5G65060.3 and AT4G38960.4 transcripts increased in *upf* mutants. The FPKM levels of the AT5G65060.3 transcript increased by 20.3, 23.9, and 25.9-fold in *upf1-5, upf3-1*, and *upf1-5 upf3-1* mutants, respectively (Figure 6C). The FPKM levels of the AT4G38950.4 transcript increased by 6.5, 2.6, and 5.8-fold in *upf1-5, upf3-1*, and *upf1-5 upf3-1* mutants, respectively (Figure 6D). We then carried out conventional RT-PCR to determine whether we could detect the aberrant transcripts identified in our RNA-seq analysis. We successfully detected amplicons using primers to specifically amplify the aberrant AT5G65060.3 and AT4G38960.4 transcripts (Figure 6E). Furthermore, the levels of AT5G65060.3 and AT4G38960.4 apparently increased in *upf* mutants, compared to wild-type plants, suggesting that AT5G65060.3 and AT4G38960.4 transcripts were likely substrates of NMD. These analyses suggested that *MAF3* and *BBX19* are potential targets of NMD in the regulation of flowering time in *upf* mutants.

## Discussion

This study aimed to explain the late-flowering phenotype of NMD-deficient mutants (*upf1-5, upf3-1*, and *upf1-5 upf3-1*) using next-generation RNA sequencing and qPCR analyses. We found that *FLC* mRNA levels were high in *upf* mutants and *FLC* likely mediates late flowering of *upf* mutants via possible transcriptional repression of *FT* and *SOC1*. However, *FLC* might not be the direct target of NMD, suggesting that upstream regulators of *FLC* may be responsible for the *UPF*-mediated flowering time change. Indeed, we found that the transcript levels of *MAF3* and *BBX19* increased in *upf* mutants and that they produced aberrant transcripts containing a PTC.

NMD is a conserved eukaryotic surveillance mechanism that protects cells from potential harmful effects of truncated proteins as a result of faulty transcripts with PTCs. Early transcript profiling experiments in Arabidopsis showed that transcript levels of only ~0.5% of protein-coding genes were elevated in NMD-impaired protoplasts (Kurihara et al., 2009), which suggests the involvement of NMD in global-scale regulation of Arabidopsis genes, apart from degrading aberrant transcripts. With the development of more robust sequencing technologies, especially RNA sequencing (RNA-seq), further insight into the mechanism of NMD and its targets has been achieved over the past few years (Filichkin et al., 2010; Drechsel et al., 2013). By using paired-end RNA-seq, we were able to find a large number of protein coding genes that were differentially expressed in *upf* mutants (2,254 genes in *upf1-5* mutants, 4,404 in *upf3-1* mutants, and 4,541 in *upf1-5 upf3-1* double mutants; Figures 2B,C). We found an additive effect of the *upf1-5* and *upf3-1* mutations on differentially expressed genes, which is consistent with the further delayed flowering seen in *upf1-5 upf3-1* double mutants (Arciga-Reyes et al., 2006; Drechsel et al., 2013). Our data suggested that the *upf3* mutation made a strong contribution to the differential expression of genes in the double mutants.

The transition from vegetative growth to flowering is a major developmental event in the plant life cycle. The precisely regulated timing of flowering has decisive consequences for the successful completion of the plant life cycle (Huijser and Schmid, 2011). Although a previous work suggested that NMD regulates *SOC1* transcript levels in the presence of EARLY FLOWERING 9 (ELF9) protein (Song et al., 2009), the molecular mechanism governing the late-flowering phenotype of NMD-deficient mutants has remained an unsolved puzzle. The *upf1-5* and *upf3-1* mutants have a delayed-flowering phenotype (Arciga-Reyes et al., 2006), but no mechanism explaining the late-flowering time phenotype was suggested. Here, we attempted to understand the factors responsible for the delayed flowering of *upf1-5* and *upf3-1* mutants, two major components in the NMD pathway in Arabidopsis, as well as in their double mutant plants. We found that *FLC* expression levels increased in *upf* mutants, which is consistent with a previous finding (Kurihara et al., 2009). In their report, the authors performed a whole-genome tiling array and found *FLC* as one of the 138 protein-coding transcripts whose expression levels were up-regulated (over 1.8-fold) in *upf* mutants. From our RNA-seq and qPCR analyses, we found consistent results showing that *FLC* mRNA levels increased in both *upf1-5* and *upf3-1* single mutants and in *upf1-5 upf3-1* double mutants (Figures 3A,B), which is consistent with the late-flowering phenotypes of *upf* mutants. This strongly suggests that *FLC* mediates the delayed flowering of NMD-deficient mutants, as *FLC* expression quantitatively and inversely correlates with flowering time (Michaels and Amasino, 1999; Sheldon et al., 1999). This conclusion is further supported by the vernalization responsiveness of *upf* mutants (Figure 4). As vernalization results in acceleration of flowering time via the stable repression of *FLC* through histone methylation (Searle et al., 2006), the recovery of the late-flowering phenotype of *upf* mutants suggests that *FLC* plays an important role in the determination of flowering time in the *upf* mutants.

How is *UPF*-mediated flowering time regulated? Although *FLC* mRNA levels increased in *upf* mutants, we could not find any evidence that *FLC* is a direct target of NMD (Supplementary Figure 5). Among upstream flowering time regulators that act in an either *FLC*-dependent or *FLC*-independent manners, however, we found that *MAF3* and *BBX19* mRNA levels increased in *upf* mutants (Figures 5B,C,J,K, respectively). Furthermore, we detected aberrant transcripts of *MAF3* and *BBX19* in *upf* mutants (Figure 6), suggesting that these two genes might be targets of the *UPF*-dependent regulation of flowering time. Thus we propose that the regulation of *UPF*-dependent flowering time involves at least two independent pathways. The first pathway is the *MAF3-FLC* pathway, in which *MAF3* positively regulates *FLC* expression (Gu et al., 2013) and subsequent up-regulation of *FLC* causes late flowering in *upf* mutants. The second pathway is the *BBX19-FT* pathway, which acts independently of *FLC*. BBX19 physically interacts with CO and prevents it from binding to the *FT* promoter to induce *FT* expression (Wang et al., 2014). Thus, increased *BBX19* expression likely leads to delayed flowering in *upf* mutants. The existence of multiple pathways is consistent with the flowering time changes induced by changes in *MAF3* and *BBX19* expression. Since flowering time changes caused by the alteration of expression of *MAF3* or *BBX19* were not very dramatic, either the *MAF3-FLC* pathway or the *BBX19-FT* pathway is not sufficient to explain the strong late flowering of *upf* mutants. Thus, we suggest that NMD affects multiple pathways to modulate flowering time and at least the *MAF3-FLC* and *BBX19-FT* modules play an important role in such regulation.

We propose that MAF3 and BBX19 function as major players in *UPF*-mediated delayed flowering in *upf* mutants, but it should be noted that the detection of PTCs and altered transcripts in *upf* mutants involves an element of random chance. Therefore, we cannot exclude the possibility that the *FLC* locus produced PTCs and altered transcripts, but we were not able to capture the event. Furthermore, considering that PTCs and altered transcripts can arise from every transcribed gene in the genome, given enough time, it is possible that the delayed flowering resulted from the combinatorial effects of many genes that may or may not be related to flowering. Given that disruption of NMD in the *upf1-5* and *upf3-1* mutants likely has broad effects on cellular metabolism, stress responses, and other aspects of plant cell biology, we consider that the observed delay in flowering time reflects only a small part of the disruptions occurring in these mutants. However, this phenotype provided a valuable indicator of the metabolic alterations occurring in the mutant cells and allowed us to probe the effects on the regulation of flowering time.

**Figure 7:**
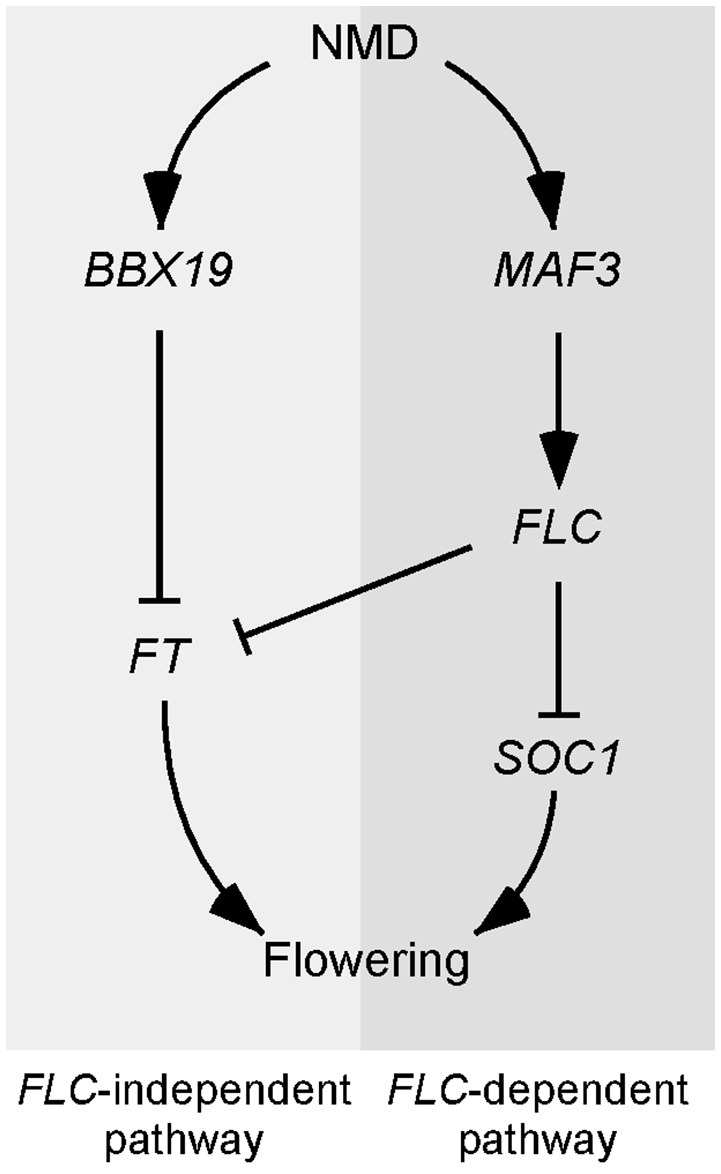
**Model explaining the late-flowering phenotype of NMD-deficient mutants**. Two independent pathways likely participate in the *UPF*-mediated regulation of flowering time. In the *FLC*-dependent pathway, *MAF3* seems to be the direct target of NMD and the up-regulation of *MAF3* in NMD-deficient mutants causes up-regulation of *FLC*, which in turn represses *FT* and *SOC1* to delay flowering. In the *FLC*-independent pathway, *BBX19* seems to be the direct target of NMD and its up-regulation suppresses *FT* expression, which eventually inhibits flowering.

Based on our results, we constructed a working model to explain the delayed flowering of *upf* mutants (Figure 7). According to this model, the regulation of *UPF*-dependent flowering time is controlled by at least two independent pathways, namely, an *FLC*-dependent pathway and an *FLC*-independent pathway. In the *FLC*-dependent pathway, *MAF3* is likely a direct substrate of NMD and aberrant *MAF3* transcripts that are not cleared out by NMD affect normal *MAF3* function, which affects *FLC*. The increase in FLC then causes the repression of flowering by direct repression of *FT* and *SOC1* by FLC. In the *FLC*-independent pathway, *BBX19* is likely a direct substrate of NMD and aberrant *BBX19* transcripts that are not removed by NMD affect normal *BBX19* function, which eventually affects *FT* expression by inhibiting binding of CO to the *FT* locus (Wang et al., 2014). Thus, reduced *FT* and *SOC1* expression is not sufficient to induce the expression of floral identity genes and hence the *upf* mutants flower later than wild-type plants.

## Author contributions

ZN analyzed the NGS data analysis, performed the experiments. MF performed the initial NGS data analysis. JA designed and supervised the study. ZN, MF, and JA wrote the manuscript.

## Funding

Our work was supported by a National Research Foundation of Korea grant funded by the Korean government (Ministry of Science, ICT, and Future Planning; 2008-0061988) to JA.

### Conflict of interest statement

The authors declare that the research was conducted in the absence of any commercial or financial relationships that could be construed as a potential conflict of interest.

## References

[B1] Abe M. Kobayashi Y. Yamamoto S. Daimon Y. Yamaguchi A. Ikeda Y.. (2005). FD, a bZIP protein mediating signals from the floral pathway integrator FT at the shoot apex. Science 309, 1052–1056. 10.1126/science.1115983 16099979

[B2] Arciga-Reyes L. Wootton L. Kieffer M. Davies B. (2006). UPF1 is required for nonsense-mediated mRNA decay (NMD) and RNAi in Arabidopsis. Plant J. 47, 480–489. 10.1111/j.1365-313X.2006.02802.x 16813578

[B3] Bastow R. Mylne J. S. Lister C. Lippman Z. Martienssen R. A. Dean C. (2004). Vernalization requires epigenetic silencing of FLC by histone methylation. Nature 427, 164–167. 10.1038/nature02269 14712277

[B4] Bernier G. Périlleux C. (2005). A physiological overview of the genetics of flowering time control. Plant Biotechnol. J. 3, 3–16. 10.1111/j.1467-7652.2004.00114.x 17168895

[B5] Bieluszewski T. Galganski L. Sura W. Bieluszewska A. Abram M. Ludwikow A.. (2015). AtEAF1 is a potential platform protein for Arabidopsis NuA4 acetyltransferase complex. BMC Plant Biol. 15:1. 10.1186/s12870-015-0461-1 25849764 PMC4358907

[B6] Bindea G. Mlecnik B. Hackl H. Charoentong P. Tosolini M. Kirilovsky A.. (2009). ClueGO: a Cytoscape plug-in to decipher functionally grouped gene ontology and pathway annotation networks. Bioinformatics 25, 1091–1093. 10.1093/bioinformatics/btp101 19237447 PMC2666812

[B7] Borner R. Kampmann G. Chandler J. Gleißner R. Wisman E. Apel K.. (2000). A MADS domain gene involved in the transition to flowering in Arabidopsis. Plant J. 24, 591–599. 10.1046/j.1365-313x.2000.00906.x 11123798

[B8] Bouche F. Lobet G. Tocquin P. Perilleux C. (2016). FLOR-ID: an interactive database of flowering-time gene networks in *Arabidopsis thaliana*. Nucleic Acids Res. 44, D1167–D1171. 10.1093/nar/gkv1054 26476447 PMC4702789

[B9] Dai Y. Li W. An L. (2016). NMD mechanism and the functions of Upf proteins in plant. Plant Cell Rep. 35, 5–15. 10.1007/s00299-015-1867-9 26400685

[B10] Deng W. Ying H. Helliwell C. A. Taylor J. M. Peacock W. J. Dennis E. S. (2011). *FLOWERING LOCUS C* (FLC) regulates development pathways throughout the life cycle of Arabidopsis. Proc. Natl. Acad. Sci. U.S.A. 108, 6680–6685. 10.1073/pnas.1103175108 21464308 PMC3081018

[B11] Drechsel G. Kahles A. Kesarwani A. K. Stauffer E. Behr J. Drewe P.. (2013). Nonsense-mediated decay of alternative precursor mRNA splicing variants is a major determinant of the Arabidopsis steady state transcriptome. Plant Cell 25, 3726–3742. 10.1105/tpc.113.115485 24163313 PMC3877825

[B12] Filichkin S. A. Mockler T. C. (2012). Unproductive alternative splicing and nonsense mRNAs: a widespread phenomenon among plant circadian clock genes. Biol. Direct 7:20. 10.1186/1745-6150-7-20 22747664 PMC3403997

[B13] Filichkin S. A. Priest H. D. Givan S. A. Shen R. Bryant D. W. Fox S. E.. (2010). Genome-wide mapping of alternative splicing in *Arabidopsis thaliana*. Genome Res. 20, 45–58. 10.1101/gr.093302.109 19858364 PMC2798830

[B14] Goff L. A. Trapnell C. Kelley D. (2012). CummeRbund: Visualization and Exploration of Cufflinks High-throughput Sequencing Data. R Package Version 2(0).

[B15] Gu X. Le C. Wang Y. Li Z. Jiang D. Wang Y.. (2013). *Arabidopsis* FLC clade members form flowering-repressor complexes coordinating responses to endogenous and environmental cues. Nat. Commun. 4:1947. 10.1038/ncomms2947 23770815 PMC3709509

[B16] He F. Li X. Spatrick P. Casillo R. Dong S. Jacobson A. (2003). Genome-wide analysis of mRNAs regulated by the nonsense-mediated and 5′ to 3′ mRNA decay pathways in yeast. Mol. Cell 12, 1439–1452. 10.1016/S1097-2765(03)00446-5 14690598

[B17] Helliwell C. A. Wood C. C. Robertson M. James Peacock W. Dennis E. S. (2006). The Arabidopsis FLC protein interacts directly *in vivo* with SOC1 and FT chromatin and is part of a high-molecular-weight protein complex. Plant J. 46, 183–192. 10.1111/j.1365-313X.2006.02686.x 16623882

[B18] Hepworth S. R. Valverde F. Ravenscroft D. Mouradov A. Coupland G. (2002). Antagonistic regulation of flowering-time gene SOC1 by CONSTANS and FLC via separate promoter motifs. EMBO J. 21, 4327–4337. 10.1093/emboj/cdf432 12169635 PMC126170

[B19] Hong S. M. Bahn S. C. Lyu A. Jung H. S. Ahn J. H. (2010). Identification and testing of superior reference genes for a starting pool of transcript normalization in Arabidopsis. Plant Cell Physiol. 51, 1694–1706. 10.1093/pcp/pcq128 20798276

[B20] Hori K. Watanabe Y. (2005). UPF3 suppresses aberrant spliced mRNA in Arabidopsis. Plant J. 43, 530–540. 10.1111/j.1365-313X.2005.02473.x 16098107

[B21] Hou X. Zhou J. Liu C. Liu L. Shen L. Yu H. (2014). Nuclear factor Y-mediated H3K27me3 demethylation of the SOC1 locus orchestrates flowering responses of *Arabidopsis*. Nat. Commun. 5:4601. 10.1038/ncomms5601 25105952

[B22] Huang N. C. Jane W. N. Chen J. Yu T. S. (2012). *Arabidopsis thaliana* CENTRORADIALIS homologue (ATC) acts systemically to inhibit floral initiation in Arabidopsis. Plant J. 72, 175–184. 10.1111/j.1365-313X.2012.05076.x 22702636

[B23] Huijser P. Schmid M. (2011). The control of developmental phase transitions in plants. Development 138, 4117–4129. 10.1242/dev.063511 21896627

[B24] Kalyna M. Simpson C. G. Syed N. H. Lewandowska D. Marquez Y. Kusenda B.. (2012). Alternative splicing and nonsense-mediated decay modulate expression of important regulatory genes in *Arabidopsis*. Nucleic acids Res. 40, 2454–2469. 10.1093/nar/gkr932 22127866 PMC3315328

[B25] Kardailsky I. Shukla V. K. Ahn J. H. Dagenais N. Christensen S. K. Nguyen J. T.. (1999). Activation tagging of the floral inducer FT. Science 286, 1962–1965. 10.1126/science.286.5446.1962 10583961

[B26] Kerényi Z. Mérai Z. Hiripi L. Benkovics A. Gyula P. Lacomme C.. (2008). Inter-kingdom conservation of mechanism of nonsense-mediated mRNA decay. EMBO J. 27, 1585–1595. 10.1038/emboj.2008.88 18451801 PMC2426726

[B27] Kertesz S. Kerenyi Z. Merai Z. Bartos I. Palfy T. Barta E.. (2006). Both introns and long 3′-UTRs operate as cis-acting elements to trigger nonsense-mediated decay in plants. Nucleic Acids Res. 34, 6147–6157. 10.1093/nar/gkl737 17088291 PMC1693880

[B28] Kobayashi Y. Kaya H. Goto K. Iwabuchi M. Araki T. (1999). A pair of related genes with antagonistic roles in mediating flowering signals. Science 286, 1960–1962. 10.1126/science.286.5446.1960 10583960

[B29] Kurihara Y. Matsui A. Hanada K. Kawashima M. Ishida J. Morosawa T.. (2009). Genome-wide suppression of aberrant mRNA-like noncoding RNAs by NMD in Arabidopsis. Proc. Natl. Acad. Sci. U.S.A. 106, 2453–2458. 10.1073/pnas.0808902106 19181858 PMC2650177

[B30] Lee H. Suh S.-S. Park E. Cho E. Ahn J. H. Kim S.-G.. (2000). The AGAMOUS-LIKE 20 MADS domain protein integrates floral inductive pathways in Arabidopsis. Genes Dev. 14, 2366–2376. 10.1101/gad.813600 10995392 PMC316936

[B31] Lee I. Amasino R. M. (1995). Effect of vernalization, photoperiod, and light quality on the flowering phenotype of Arabidopsis plants containing the FRIGIDA gene. Plant Physiol. 108, 157–162. 10.1104/pp.108.1.157 12228459 PMC157316

[B32] Li D. Liu C. Shen L. Wu Y. Chen H. Robertson M.. (2008). A repressor complex governs the integration of flowering signals in Arabidopsis. Dev. Cell 15, 110–120. 10.1016/j.devcel.2008.05.002 18606145

[B33] Maquat L. E. (2005). Nonsense-mediated mRNA decay in mammals. J. Cell Sci. 118, 1773–1776. 10.1242/jcs.01701 15860725

[B34] Mendell J. Sharifi N. Meyers J. Martinez-Murillo F. Dietz H. (2004). Erratum: nonsense surveillance regulates expression of diverse classes of mammalian transcripts and mutes genomic noise. Nat. Genet. 36, 1238–1238. 10.1038/ng1104-1238c 15448691

[B35] Michaels S. D. (2009). Flowering time regulation produces much fruit. Curr. Opin. Plant Biol. 12, 75–80. 10.1016/j.pbi.2008.09.005 18938104 PMC2644822

[B36] Michaels S. D. Amasino R. M. (1999). *FLOWERING LOCUS C* encodes a novel MADS domain protein that acts as a repressor of flowering. Plant Cell 11, 949–956. 10.1105/tpc.11.5.949 10330478 PMC144226

[B37] Mimida N. Goto K. Kobayashi Y. Araki T. Ahn J. H. Weigel D.. (2001). Functional divergence of the TFL1-like gene family in Arabidopsis revealed by characterization of a novel homologue. Genes Cells 6, 327–336. 10.1046/j.1365-2443.2001.00425.x 11318875

[B38] Nyikó T. Kerényi F. Szabadkai L. Benkovics A. H. Major P. Sonkoly B.. (2013). Plant nonsense-mediated mRNA decay is controlled by different autoregulatory circuits and can be induced by an EJC-like complex. Nucleic Acids Res. 41, 6715–6728. 10.1093/nar/gkt366 23666629 PMC3711448

[B39] Rayson S. Arciga-Reyes L. Wootton L. Zabala M. D. T. Truman W. Graham N.. (2012). A role for nonsense-mediated mRNA decay in plants: pathogen responses are induced in *Arabidopsis thaliana* NMD mutants. PLoS ONE 7:e31917. 10.1371/journal.pone.0031917 22384098 PMC3284524

[B40] Rehwinkel J. Letunic I. Raes J. Bork P. Izaurralde E. (2005). Nonsense-mediated mRNA decay factors act in concert to regulate common mRNA targets. RNA 11, 1530–1544. 10.1261/rna.2160905 16199763 PMC1370837

[B41] Riehs N. Akimcheva S. Puizina J. Bulankova P. Idol R. A. Siroky J.. (2008). Arabidopsis SMG7 protein is required for exit from meiosis. J. Cell Sci. 121, 2208–2216. 10.1242/jcs.027862 18544632

[B42] Riehs-Kearnan N. Gloggnitzer J. Dekrout B. Jonak C. Riha K. (2012). Aberrant growth and lethality of Arabidopsis deficient in nonsense-mediated RNA decay factors is caused by autoimmune-like response. Nucleic Acids Res. 40, 5615–5624. 10.1093/nar/gks195 22379136 PMC3384318

[B43] Searle I. He Y. Turck F. Vincent C. Fornara F. Krober S.. (2006). The transcription factor FLC confers a flowering response to vernalization by repressing meristem competence and systemic signaling in Arabidopsis. Genes Dev. 20, 898–912. 10.1101/gad.373506 16600915 PMC1472290

[B44] Sheldon C. C. Burn J. E. Perez P. P. Metzger J. Edwards J. A. Peacock W. J.. (1999). The FLF MADS box gene: a repressor of flowering in Arabidopsis regulated by vernalization and methylation. Plant Cell 11, 445–458. 10.1105/tpc.11.3.445 10072403 PMC144185

[B45] Shi C. Baldwin I. T. Wu J. (2012). Arabidopsis plants having defects in nonsense-mediated mRNA decay factors UPF1, UPF2, and UPF3 show photoperiod-dependent phenotypes in development and stress responses. J. Integr. Plant Biol. 54, 99–114. 10.1111/j.1744-7909.2012.01093.x 22353561

[B46] Song H.-R. Song J.-D. Cho J.-N. Amasino R. M. Noh B. Noh Y.-S. (2009). The RNA binding protein ELF9 directly reduces suppressor of overexpression of CO1 transcript levels in Arabidopsis, possibly via nonsense-mediated mRNA decay. Plant Cell 21, 1195–1211. 10.1105/tpc.108.064774 19376936 PMC2685614

[B47] Spitzer M. Wildenhain J. Rappsilber J. Tyers M. (2014). BoxPlotR: a web tool for generation of box plots. Nat. Methods 11, 121–122. 10.1038/nmeth.2811 24481215 PMC3930876

[B48] Suter L. Rüegg M. Zemp N. Hennig L. Widmer A. (2014). Gene regulatory variation mediates flowering responses to vernalization along an altitudinal gradient in Arabidopsis. Plant Physiol. 166, 1928–1942. 10.1104/pp.114.247346 25339407 PMC4256870

[B49] Trapnell C. Roberts A. Goff L. Pertea G. Kim D. Kelley D. R.. (2012). Differential gene and transcript expression analysis of RNA-seq experiments with TopHat and Cufflinks. Nat. Protoc. 7, 562–578. 10.1038/nprot.2012.016 22383036 PMC3334321

[B50] Wang C.-Q. Guthrie C. Sarmast M. K. Dehesh K. (2014). BBX19 interacts with CONSTANS to repress *FLOWERING LOCUS T* transcription, defining a flowering time checkpoint in Arabidopsis. Plant Cell 26, 3589–3602. 10.1105/tpc.114.130252 25228341 PMC4213167

[B51] Xu M. Y. Zhang L. Li W. W. Hu X. L. Wang M.-B. Fan Y. L.. (2013). Stress-induced early flowering is mediated by miR169 in *Arabidopsis thaliana*. J. Exp. Bot. 65, 89–101. 10.1093/jxb/ert353 24336445

[B52] Yoine M. Nishii T. Nakamura K. (2006). Arabidopsis UPF1 RNA helicase for nonsense-mediated mRNA decay is involved in seed size control and is essential for growth. Plant Cell Physiol. 47, 572–580. 10.1093/pcp/pcj035 16540482

[B53] Zacharaki V. Benhamed M. Poulios S. Latrasse D. Papoutsoglou P. Delarue M.. (2012). The Arabidopsis ortholog of the YEATS domain containing protein YAF9a regulates flowering by controlling H4 acetylation levels at the FLC locus. Plant Sci. 196, 44–52. 10.1016/j.plantsci.2012.07.010 23017898

[B54] Zeevaart J. A. (2008). Leaf-produced floral signals. Curr. Opin. Plant Biol. 11, 541–547. 10.1016/j.pbi.2008.06.009 18691931

