# Supplementary material for: Possible Role of MADS AFFECTING FLOWERING 3 and B-BOX DOMAIN PROTEIN 19 in Flowering Time Regulation of Arabidopsis Mutants with Defects in Nonsense-Mediated mRNA Decay
Source: Front Plant Sci. 2017 Feb 14;8:191. doi: 10.3389/fpls.2017.00191 (PMC5306368; doi:10.3389/fpls.2017.00191)
Supplement: Supplementary file 2 [file DataSheet1.docx]

Supplementary Material

Possible role of MADS AFFECTING FLOWERING 3 and *B-BOX DOMAIN PROTEIN 19* in flowering time regulation of Arabidopsis mutants with defects in nonsense-mediated mRNA decay

Zeeshan Nasim^1^, Muhammad Fahim^2^, Ji Hoon Ahn^1*^

# * Correspondence: Ji Hoon Ahn: jahn@korea.ac.kr

# Supplementary Data

# Supplementary Figures and Tables

## Supplementary Figures


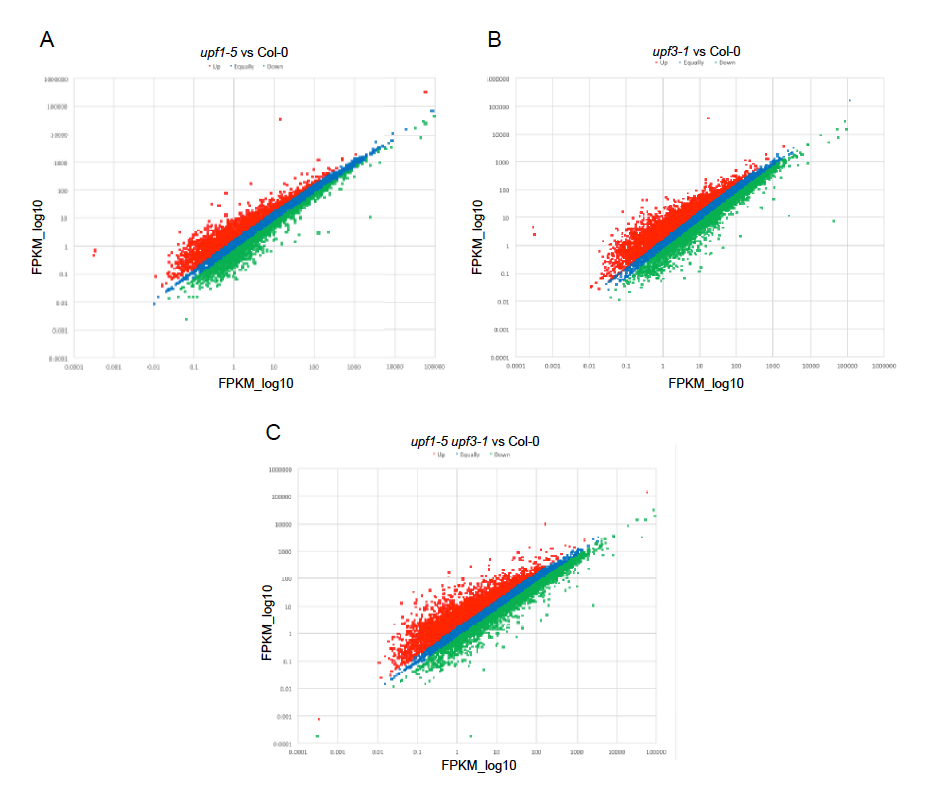


**Supplementary Figure 1.** **Scatter plots of gene expression in** ***upf1-5, upf3-1,* and *upf1-5 upf3-1* mutants versus wild-type plants.** Scatter plot representing the DEGs between *upf1-5* mutants and Col-0 plants (A), between *upf3-1* mutants and Col-0 plants (B), and between *upf1-5 upf3-1* double mutants and Col-0 plants (C). Red dots represent up-regulated genes and green dots represent down-regulated genes in the mutants, compared to wild-type plants, whereas genes with similar expression to wild-type plants are shown as blue dots. Note that the *upf3-1* and *upf1-5* *upf3-1* double mutants have more red dots and green dots and share similar distribution patterns of these dots, compared to *upf1-5* mutants, suggesting a possibility that *upf3* has a stronger effect than *upf1*.

**
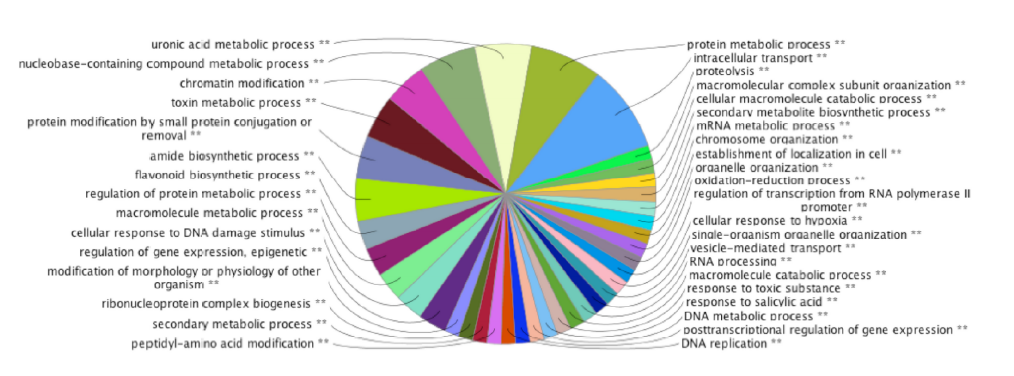
**

**Supplementary Figure 2. Gene Ontology (GO) analysis for the DEGs in *upf* mutants.** GO analysis for biological process was performed using the ClueGO plugin of Cytoscape with a *p*-value threshold of 0.05. Double asterisks (**) show that the GO term is significant (*p* value <0.001).


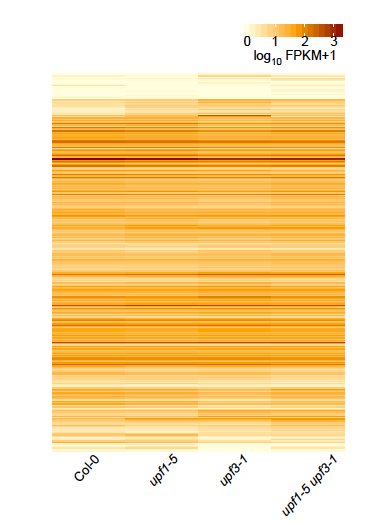


**Supplementary Figure 3.** Heatmap showing expression profiles of genes that regulate flowering time between Col-0 and *upf* mutants.


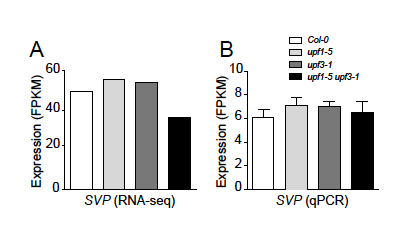


**Supplementary Figure 4. *SVP* mRNA expression levels in *upf1-5, upf3-1,* and *upf1-5 upf3-1* mutants.** **(A, B)** FPKM levels determined by RNA-seq (A) and relative expression levels determined by qPCR (B) of *SVP* in *upf1-5*, *upf3-1*, *upf1-5 upf3-1*, and wild-type plants. Note no clear differences between wild-type plants and *upf* mutants.


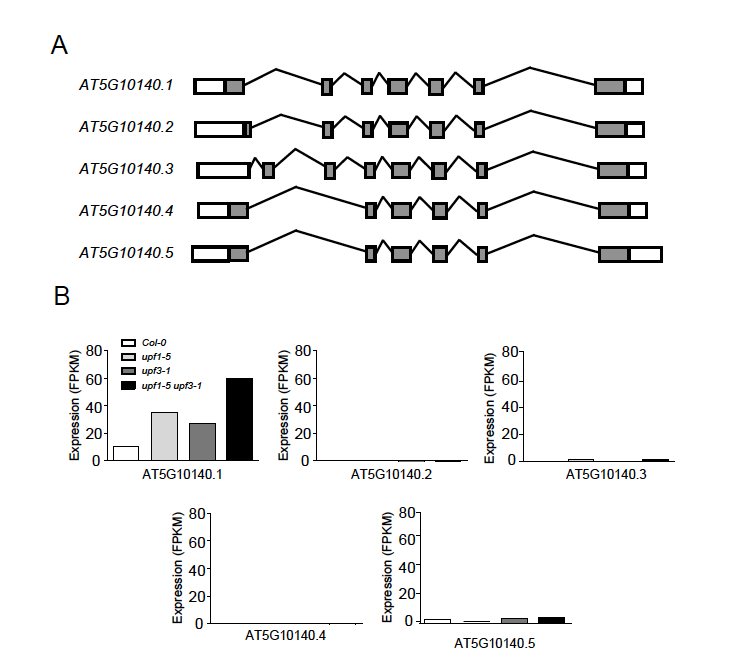


**Supplementary Figure 5. Structures and FPKM values of alternatively spliced isoforms of *FLC*. (A)** Structures of alternatively spliced isoforms of *FLC* detected by RNA-seq analysis. Note that the AT5G10140.5 transcript is a newly found isoform in our RNA-seq analysis. A box indicates an exon. The coding sequence is indicated in grey. **(B)** FPKM levels of alternative spliced isoforms of *FLC*. The levels of the AT5G10140.1 transcript, which is the predominantly occurring transcript of *FLC*, increased in *upf* mutants. However, none of the isoforms showed apparent changes in their expression levels.

**
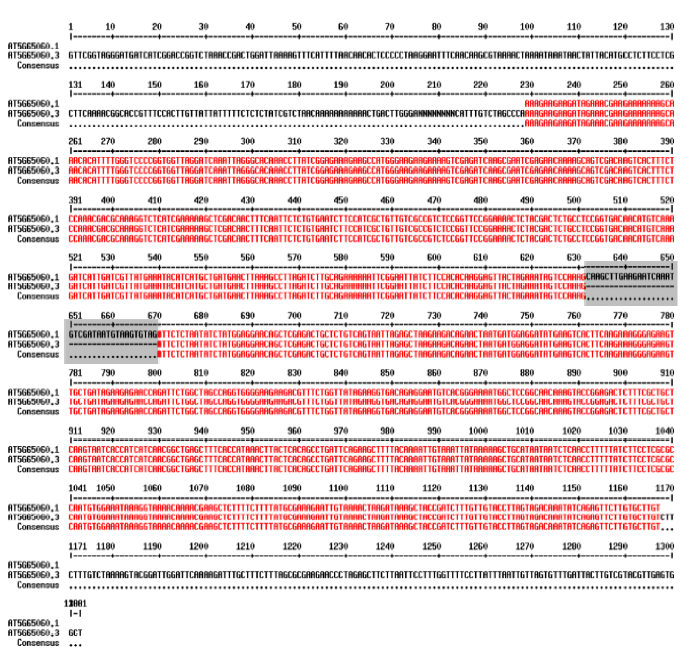
**

**Supplementary Figure 6. Alignment of cDNA sequence between the normal and aberrant transcripts of *MAF3*.** Sequence alignment shows a long 5′ and 3′ UTR in the aberrant *MAF3* transcript as well as deletion of 38 nucleotides from the start of the 4^th^ exon.

**
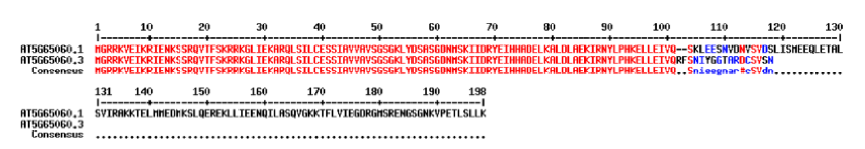
**

**Supplementary Figure 7. Protein sequence alignment of the *in silico* translated *MAF3* normal and truncated proteins.** Due to the skipping of a short exonic sequence in the aberrant transcript, the translational frame is changed. This frameshift results in a premature termination codon at the end of the 4^th^ exon as highlighted in Figure 9.

**
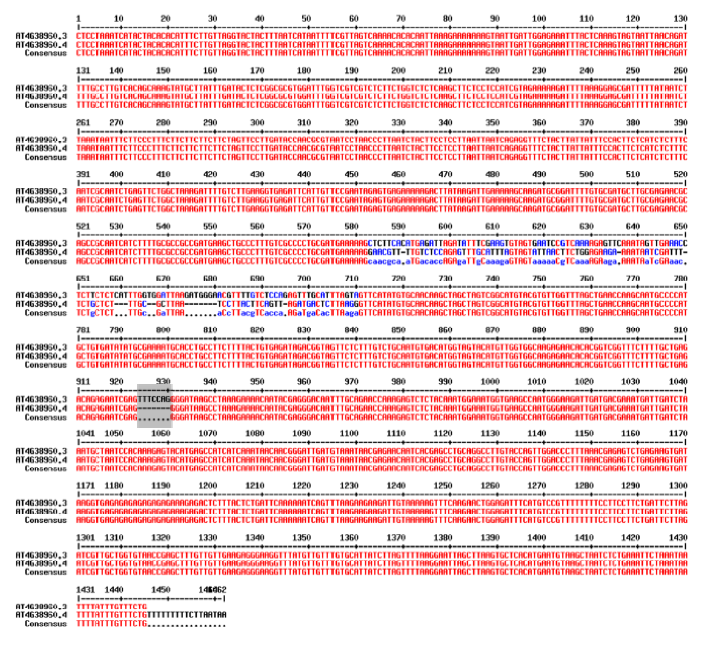
**

**Supplementary Figure 8. cDNA sequence alignment of *BBX19* normal and aberrant transcripts.** The aberrant transcript has a very long 5′ UTR and a slightly long 3′ UTR. However, there are only seven bases differences between the coding regions of the two transcripts.


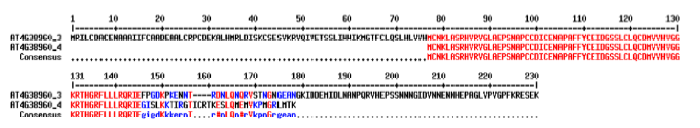


**Supplementary Figure 9. Protein sequence alignment of the *in silico* translated *BBX19* normal and truncated proteins.** Skipping of seven bases in the aberrant transcript results in a truncated protein due to a premature termination codon in exon 6.

## Supplementary Tables

**SUPPLEMENTARY TABLE 1| All DEGs between Arabidopsis wild-type and *upf* mutant plants (Excel file)**

**SUPPLEMENTARY TABLE 2 | Changes in expression levels of *FLC*, *FT*, and *SOC1* in *upf* mutants compared to wild-type plants identified from RNA-seq analysis**

| **Gene** | ***upf1-5*** | ***upf3-1*** | ***upf1-5 upf3-1*** | **Expression** | **Role** |
| --- | --- | --- | --- | --- | --- |
| *FLC* | 2.92 | 2.45 | 5.09 | Upregulated | Repressor |
| *FT* | 1.1 | 2.63 | 5.15 | Downregulated | Promoter |
| *SOC1* | 1.71 | 4.56 | 6.33 | Downregulated | Promoter |

**SUPPLEMENTARY TABLE 3 | Expression of *FLC* regulators in *upf* mutants**

| ***FLC* regulators** | | **Expression level (FPKM)** | | | |
| --- | --- | --- | --- | --- | --- |
| **Gene** | **ID** | **Col-0** | ***upf1-5*** | ***upf3-1*** | ***upf1-5 upf3-1*** |
| *MEA* | AT1G02580 | 0.129178 | 0.0512073 | 0 | 0 |
| *ATX2* | AT1G05830 | 23.1058 | 18.7241 | 13.6432 | 17.7578 |
| *DRIP1* | AT1G06770 | 7.65075 | 6.10232 | 6.50049 | 5.73224 |
| *UBC1* | AT1G14400 | 143.17 | 135.257 | 111.586 | 140.792 |
| *ARP4* | AT1G18450 | 30.647 | 27.1342 | 21.404 | 22.208 |
| *SUF4* | AT1G30970 | 48.338 | 38.1952 | 39.4848 | 44.4735 |
| *TRO* | AT1G51450 | 7.14641 | 5.90069 | 6.56186 | 5.47965 |
| *NF-YA5* | AT1G54160 | 0.774942 | 2.70766 | 4.34387 | 6.76569 |
| *HUB2* | AT1G55250 | 11.3025 | 11.2769 | 12.1027 | 12.2768 |
| *VIP5* | AT1G61040 | 9.70643 | 8.60149 | 9.00923 | 10.9338 |
| *AGL27* | AT1G77080 | 59.4768 | 62.3285 | 49.0029 | 67.1516 |
| *EFS* | AT1G77300 | 14.9466 | 10.3532 | 13.7834 | 13.4171 |
| *VIP2* | AT1G79730 | 23.0783 | 18.9302 | 17.555 | 21.0549 |
| *UBC2* | AT2G02760 | 170.99 | 126.205 | 105.617 | 115.601 |
| *VIP6* | AT2G06210 | 13.4464 | 11.6848 | 10.4221 | 12.6104 |
| *ABH1* | AT2G13540 | 16.9141 | 18.8922 | 17.6419 | 16.3532 |
| *TAF14* | AT2G18000 | 1.79365 | 3.63833 | 5.66972 | 3.87349 |
| *MSI4* | AT2G19520 | 31.0488 | 31.303 | 22.4423 | 27.2013 |
| *CLF* | AT2G23380 | 5.4645 | 9.64878 | 8.31671 | 9.23908 |
| *FLX* | AT2G30120 | 11.4432 | 11.4118 | 14.743 | 10.361 |
| *DRIP2* | AT2G30580 | 8.55601 | 7.79462 | 8.27327 | 8.15445 |
| *ATX1* | AT2G31650 | 3.99493 | 3.23569 | 3.72175 | 3.3803 |
| *FES1* | AT2G33835 | 5.96277 | 4.70388 | 4.80045 | 5.55784 |
| *HUB1* | AT2G44950 | 13.2921 | 13.1544 | 16.2285 | 15.0788 |
| *NF-YA2* | AT3G05690 | 12.1669 | 17.7485 | 25.5983 | 39.5357 |
| *FLD* | AT3G10390 | 7.75263 | 6.76804 | 6.36533 | 7.19303 |
| *PIE1* | AT3G12810 | 5.4464 | 6.08293 | 7.63355 | 9.23304 |
| *VRN1* | AT3G18990 | 35.1995 | 42.3355 | 47.7726 | 39.7363 |
| *FIE* | AT3G20740 | 22.7817 | 22.1571 | 19.6324 | 18.5383 |
| *CDC37* | AT3G22590 | 17.7706 | 15.7975 | 16.6398 | 16.2113 |
| *VIL1* | AT3G24440 | 9.73492 | 8.77597 | 9.84521 | 10.0877 |
| *SSRP1* | AT3G28730 | 24.0854 | 27.3136 | 28.4865 | 26.2933 |
| *ARP6* | AT3G33520 | 31.0528 | 22.3002 | 16.1585 | 19.9849 |
| *WDR5A* | AT3G49660 | 5.621 | 6.58563 | 5.97215 | 4.44177 |
| *FRI* | AT4G00650 | 6.30595 | 6.72007 | 5.37354 | 6.44114 |
| *LIF2* | AT4G00830 | 32.0321 | 38.6865 | 52.1438 | 50.8398 |
| *EZA1* | AT4G02020 | 14.3211 | 12.8181 | 12.2475 | 14.6371 |
| *SPT16* | AT4G10710 | 16.4591 | 14.5364 | 13.4066 | 15.6533 |
| *VRN2* | AT4G16845 | 22.6044 | 21.9715 | 28.5491 | 24.3335 |
| *VIP3* | AT4G29830 | 16.4014 | 16.6139 | 10.5608 | 11.5122 |
| *VIL2* | AT4G30200 | 37.916 | 36.6584 | 39.8865 | 42.6639 |
| *EMF1* | AT5G11530 | 5.91102 | 5.61114 | 5.01103 | 6.02667 |
| *FRL1* | AT5G16320 | 3.3422 | 3.24954 | 3.29254 | 3.53552 |
| *LHP1* | AT5G17690 | 12.3005 | 12.3936 | 13.0325 | 12.6662 |
| *SWC6* | AT5G37055 | 16.0014 | 11.2451 | 7.80742 | 13.4503 |
| *ATXR7* | AT5G42400 | 7.43003 | 7.47438 | 8.68477 | 9.33793 |
| *CBP20* | AT5G44200 | 33.8321 | 36.7757 | 30.759 | 30.7696 |
| *TAF14B* | AT5G45600 | 25.2525 | 26.3067 | 29.9126 | 25.8233 |
| *EMF2* | AT5G51230 | 18.0143 | 19.7084 | 23.6052 | 19.2168 |
| *MSI1* | AT5G58230 | 34.8649 | 30.3245 | 25.0074 | 24.7444 |
| *HDA5* | AT5G61060 | 15.5962 | 13.2554 | 12.1503 | 16.0088 |
| *VIP4* | AT5G61150 | 22.6619 | 20.7502 | 18.4276 | 22.1865 |
| *FLXL4* | AT5G61920 | 2.07104 | 2.05155 | 2.62799 | 2.12105 |
| *HDA6* | AT5G63110 | 12.4057 | 14.2996 | 11.3432 | 13.9833 |
| *AGL31* | AT5G65050 | 63.1507 | 71.5494 | 73.9748 | 70.0301 |
| *MAF3* | AT5G65060 | 17.0596 | 50.698 | 48.2249 | 55.5121 |
| *MAF4* | AT5G65070 | 12.5173 | 11.2117 | 5.76714 | 8.53842 |
| *MAF5* | AT5G65080 | 7.90947 | 7.6359 | 2.42409 | 2.76589 |

**SUPPLEMENTARY TABLE 4 | Primers used in this study**

| **Gene** | **Primer** | **Used for** | **Sequence (5' to 3')** | **Orientation** |
| --- | --- | --- | --- | --- |
| *ATC* | JH6382 | qPCR | AGAGCACTTGCACTGGATTG | Sense |
|  | JH6383 |  | TACGGGTCTGCTTGAACAAC | Antisense |
| *BBX19* | JH11572 | qPCR | ACGAGGGACAATTTGCAGAACC | Sense |
|  | JH11573 |  | TTCCCATTGGCTTCACCATTTCC | Antisense |
|  | JH11574 | splice variant | CCACTTCTCATCTCTTTCAATCGC | Sense |
|  | JH11575 |  | CTGAAGTAAGGATTAAGCGCAAAG | Antisense |
| *FLC* | JH10793 | qPCR | TGTGAGTATCGATGCTCTTGTTCA | Sense |
|  | JH10794 |  | TTCAACATGAGTTCGGTCTTCTTG | Antisense |
| *FT* | JH6488 | qPCR | AGGCCTTCTCAGGTTCAAAACAAGC | Sense |
|  | JH6489 |  | TGCCAAAGGTTGTTCCAGTTGTAGC | Antisense |
| *MAF3* | JH4788 | qPCR | TGGCTCCGGCAACAAAGTACC | Sense |
|  | JH4797 |  | CACATTGGCGCGAGGAAGAT | Antisense |
|  | JH11554 | splice variant | AGCCACTCAACGTACGACAAGT | Antisense |
| *NF-YA2* | JH11567 | qPCR | CGCACTTAGCTTTCTCACTTG | Sense |
|  | JH11568 |  | TGATGCATATATGGCTTGCGGCATC | Antisense |
| *NF-YA5* | JH11561 | qPCR | CCAACGGCGCTGCCACATTTAG | Sense |
|  | JH11562 |  | ATGAAGGTACGGTTTACGGC | Antisense |
| *PP2AA3* | JH6505 | qPCR | GCGGTTGTGGAGAACATGATACG | Sense |
|  | JH6506 |  | GAACCAAACACAATTCGTTGCTG | Antisense |
|  | JH11608 | RT-PCR | AGCCATTGTAGAACTTGCTG  CTATATGCTGCATTGCCCAT | Sense  Antisense |
|  | JH11609 |  |  |  |
| *SOC1* | JH11609 | qPCR | CTATATGCTGCATTGCCCAT | Antisense |
|  | JH10513 |  | TCTTGCATATTGGAGCTGGC | Antisense |
| *Sand* | JH7588 | qPCR | TTGATCCACTTGCAGACAAGGC | Sense |
|  | JH7589 |  | TACCCTTTGGCACACCTGATTG | Antisense |
| *TAF14* | JH11556 | qPCR | TGTCTGAAGCTGATGCGTCTAT | Sense |
|  | JH11557 |  | TCTCCGGGTTCAGCTTTAAGA | Antisense |
| *UPF1* | AtUPF1-1F | genotyping | ACAATCCAAATCTTCAGTCTCA | Sense |
|  | AtUPF1-2R |  | AGAGACAACAAAATCATGTCG | Antisense |
| *UPF3* | AtUPF3-4F | genotyping | ACTTCTATTGTTGATCTCTGG | Sense |
|  | AtUPF3-6R |  | ATGCTGTTCCGGTTGTGGTGG | Antisense |
| T-DNA | LBb1.3 | genotyping | ATTTTGCCGATTTCGGAAC |  |
